# Seven tesla MRI reveals amygdala and hippocampal subfield atrophy in dementia with Lewy bodies

**DOI:** 10.1186/s13195-026-02091-8

**Published:** 2026-05-27

**Authors:** Elizabeth McKiernan, Peter Swann, Elijah Mak, Maria Prats-Sedano, George Savulich, Allison Bentley, Guy Williams, Li Su, John T. O’Brien

**Affiliations:** 1 Department of Psychiatry, University of Cambridge, Cambridge Biomedical Campus, Cambridge, CB2 0SL UK; 2 Mayo Clinic, Rochester, MN 55905 USA; 3 Wolfson Brain Imaging Centre, University of Cambridge, Cambridge Biomedical Campus, Cambridge, CB2 0SL UK; 4 Sheffield Institute for Translational Neuroscience, University of Sheffield, Sheffield, S10 2HQ UK

**Keywords:** Dementia with Lewy bodies, Alzheimer’s disease, Seven tesla MRI, Hippocampus, Amygdala, Subfield volumes

## Abstract

The 7T-DLB study investigated whether atrophy of individual hippocampal and amygdala subfields is present in dementia with Lewy bodies (DLB) as well as in Alzheimer’s disease (AD) in which it has previously been established. MRI at standard clinical and research field strengths may have insufficient resolution to reveal the presence of atrophy in these regions in DLB; seven tesla MRI is ideally placed to examine subtle volumetric differences in these brain areas due to increased signal to noise ratio and tissue contrast.

In this prospective, cross-sectional, seven tesla imaging study, 20 participants with probable DLB underwent MRI, clinical assessment, and cognitive testing, alongside a patient comparison group of 25 participants with probable AD, and 19 healthy controls. Scans were segmented using FreeSurfer automated pipelines. Hippocampal and amygdala subfield volumes were compared between diagnostic groups using a generalised linear model with age, sex, education, and total intracranial volume as covariates. Associations between volumes and cognition were examined using Kendall’s tau. Exploratory analyses investigated associations between subfield volumes and visual hallucinations scores, and in a sub-set of 41 participants (DLB = 13, AD = 17, controls = 11), with plasma ptau-217 concentrations.

Four (of 13) hippocampal subfields (molecular layer, subiculum, presubiculum, and entorhinal cortex) and five (of nine) amygdala subfields (accessory basal, central, medial and cortical nuclei, and anterior amygdaloid area) were significantly smaller in DLB compared to controls (p_FDR_<0.05). In DLB, significant correlations between subfield volumes and Addenbrookes Cognitive Examination Scores were found for seven hippocampal subfields (Cornu Ammonis (CA)1, CA2/3, CA4, molecular layer, dentate gyrus, hippocampal amygdala transition area, and presubiculum), and five amygdala subfields (basal, accessory basal, central, cortical, and cortico-amygdala transition area) (all p_FDR_<0.05). Exploratory analyses suggest that amygdala subfield volumes may be associated with visual hallucinations, though these provisional findings require replication.

We conclude that structural seven tesla MRI reveals volumetric differences in individual hippocampal and amygdala subfields between DLB and controls, as well as between AD and controls where they have previously been described. Atrophy of individual subfields may potentially help explain the presence of cognitive and non-cognitive symptoms in DLB.

**Supplementary Information:**

The online version contains supplementary material available at 10.1186/s13195-026-02091-8.

## Introduction

Despite its position as the second commonest form of neurodegenerative dementia [1], dementia with Lewy bodies (DLB) is relatively poorly understood, with little research focusing on its pathophysiology or symptomatology in comparison with Alzheimer’s disease (AD) or Parkinson’s disease, to which it is closely related [2]. In contrast to AD, which typically presents with amnesic cognitive deficits, DLB more commonly presents with complex neuropsychiatric symptoms, including complex visual hallucinations, cognitive fluctuations, rapid eye movement (REM) sleep behaviour disorder (RBD) and parkinsonism [3].

In DLB, atrophy is found in frontal, parietal, lateral and medial temporal brain regions compared to controls [4], however, in the cortex these volumetric grey matter differences are less severe than in AD especially within the medial temporal lobe [5] Subcortical atrophy may be greater in DLB than in AD [6]; a focused pattern of atrophy including in the dorsal brain stem [7], dorsal midbrain, hypothalamus and substantia innominate [8] has been described, which may differentiate DLB from AD. Medial temporal lobe atrophy is found in DLB compared to controls but is less common and less severe than in AD [9], wherein disproportionate medial temporal lobe atrophy, particularly involving the hippocampal formation, is supportive of an AD diagnosis [10], subsequently relative sparing of the medial temporal lobe (compared to AD, the primary differential diagnosis [11]) is a supportive biomarker for a diagnosis of probable DLB [3].

However, the medial temporal lobe is not uniform but is a complex structure which can be segmented according to its cytoarchitecture [12]. It is composed of functionally diverse subfields which contribute in different ways to the hippocampus’ roles in episodic memory and navigational function [13]. Although challenging, due to practical limits on spatial resolution in vivo, some subfields have been delineated at 1.5 and 3T, with some studies suggesting that patterns of subfield atrophy may differ between AD and DLB; for example, atrophy of the cornu Ammonis (CA) subfields 1 and 2, and atrophy of the subiculum have been reported in AD compared to controls [14] and, in comparative studies, it has been reported that CA1, subiculum [15], fimbria and fissure [16] are relatively preserved in DLB compared to AD, while the parahippocampus and perirhinal cortex are not [15]. Other groups have reported the preservation of all subfields in DLB compared to AD [17], however, it has also been reported that comparatively severe CA2/3 and dentate gyrus/CA4 atrophy (with respect to atrophy of other subfields) may distinguish DLB from other dementias [17]. In pathological series, anti-ubiquitin staining (which is sensitive to cortical Lewy bodies) has shown the specific involvement of the CA2/3 region in DLB [18], but this does not appear to result in MR-visible atrophy in vivo [19]. Therefore, although it is apparent that the medial temporal lobe as a whole is less affected in DLB than in AD, the evidence regarding individual subfields is sparse and mixed; there may be changes to individual subfields that are not readily measurable at lower MR field strengths.

The amygdala, another complex medial temporal lobe structure, traditionally implicated in the processing of fear, emotion and social cognition [20], is a major site of Lewy body pathology in DLB [21] and in AD, where amygdala only Lewy bodies are present in around 40–60% of cases [22]. The amygdala may be of particular importance in DLB not least due to the role it may play in the propagation of Alzheimer and Lewy body co-pathology [23]. Atrophy of the whole amygdala is reported on MRI in DLB compared to controls [24] and antemortem MRI amygdala atrophy has been found to inversely associate with amygdala Lewy bodies on histopathological examination [25]. In AD, whole amygdala atrophy is also reported, and it has been proposed that this may be associated with psychiatric symptoms in AD such as anxiety and apathy [26]. Changes in amygdala functional connectivity have also been reported in AD and found to associate with markers of cognition [27], suggesting that functional as well as structural changes in the amygdala may be implicated in the production of symptoms. Outside of the dementia literature, the amygdala has been implicated in the production of visual and auditory hallucinations in psychosis [28, 29], which is intriguing given the prevalence of complex visual hallucinations in DLB [30]. Amygdala segmentation has not previously been reported at 7T in DLB; in AD, findings at 7T suggest that individual nuclei may play differential roles in the genesis of the disease, with one group reporting that atrophy of the lateral and accessory basal amygdala predicted conversion of mild cognitive impairment to AD [19]. A relatively recent resurgence in interest in the structure and function of the amygdala in AD and DLB may be due to advances in imaging technology [31].

Seven tesla (7T) MRI provides the opportunity to investigate subfield volumes in more detail than has previously been possible at lower field strengths; several studies have demonstrated the benefits of using this method to investigate hippocampal subfield volumes in AD populations [32]. Imaging parameters are altered as MR static field strength (B_0_) increases from 3 to 7 tesla. Signal to noise ratio (SNR) increases in proportion with the increase in B_0_, indirectly impacting image resolution which increases by around 30% [33]. Increases in tissue contrast to noise ratio, and sensitivity to susceptibility, and the opportunity for smaller voxel sizes and reduced scan acquisition times may also result in images better suited to the investigation of small brain structures [33]. At 7T volumetric changes in individual subfields of the hippocampus [32] and amygdala [34] have been reported in AD and mild cognitive impairment of Alzheimer’s type, and associations between subfield volumes and measures of cognitive performance reported, but no studies have yet examined these subfields in a DLB population at 7T.

The 7T-DLB study set out to explore the utility of 7T MRI for the investigation of DLB pathology. A disease comparison group with AD and a similarly aged healthy control group without subjective or objective cognitive disorder were included for comparison. The primary aim of this paper is to compare hippocampal and amygdala subfield volumes between DLB and controls. Differences in subfield volumes between AD and controls, and between DLB and AD were also examined. Relationships between subfield volumes and cognition were examined, and, in exploratory analyses, between subfield volumes and measures of a key DLB symptom of visual hallucinations.

## Materials and methods

### Participants

Participants were recruited, interviewed and scanned within the 7T-DLB study (ethics approval reference 16-EE-0418). Included were 20 participants who met the consensus criteria for probable DLB [3], 25 who met the consensus criteria for probable AD [10], and 20 cognitively healthy control participants. Participants were recruited from Cambridgeshire and Peterborough NHS Foundation Trust, Cambridge University Hospitals NHS Foundation Trust, Essex Partnership University NHS Trust, Norfolk, Suffolk NHS Foundation Trust and via the Join Dementia Research online portal (https://www.joindementiaresearch.nihr.ac.uk). The sample size of 20 participants per group to detect a difference in medial temporal lobe subfield volumes between DLB and controls was determined based on data from 7T MRI studies of AD as there were no published 7T studies in DLB to properly inform a power calculation. Details of the power calculation can be found in the Supplementary materials.

### Clinical and cognitive testing

Participants completed a battery of cognitive tests which included the Addenbrookes Cognitive Examination-III [35] and a visual hallucinations interview (derived from previously validated scales [36, 37]).

### Blood collection, processing and analysis

In a subset of 41 participants (DLB = 13, AD = 17, controls = 11) blood samples were obtained by venepuncture and collected in Ethylenediaminetetraacetic acid tubes. They were centrifuged to isolate plasma, aliquoted and stored at − 70/80°C until analysis with the Alzpath simoa ptau-217 assay at UK Dementia Research Institute biomarker factor, University College London [38]. The measurement of plasma ptau-217 concentrations was intended for additional exploratory analyses and was not used to support diagnostic characterisation.

### Neuroimaging

#### Image acquisition

Imaging data was acquired on 7T Siemens MAGNETOM Terra (Siemens Healthineers, Erlangen, Germany) at the Wolfson Brain Imaging Centre (University of Cambridge) as part of the 7T-DLB multi-model MRI protocol. Structural imaging was acquired from a structural magnetization-prepared 2 rapid acquisition gradient echo (MP2RAGE) sequence. Repetition time=4300ms, echo time = 1.99ms, 224 slices, flip angle 1 = 5°, FA2 = 6°, voxel size = 0.75 × 0.75 × 0.75mm^3^.

#### Image processing

**Fig. 1:**
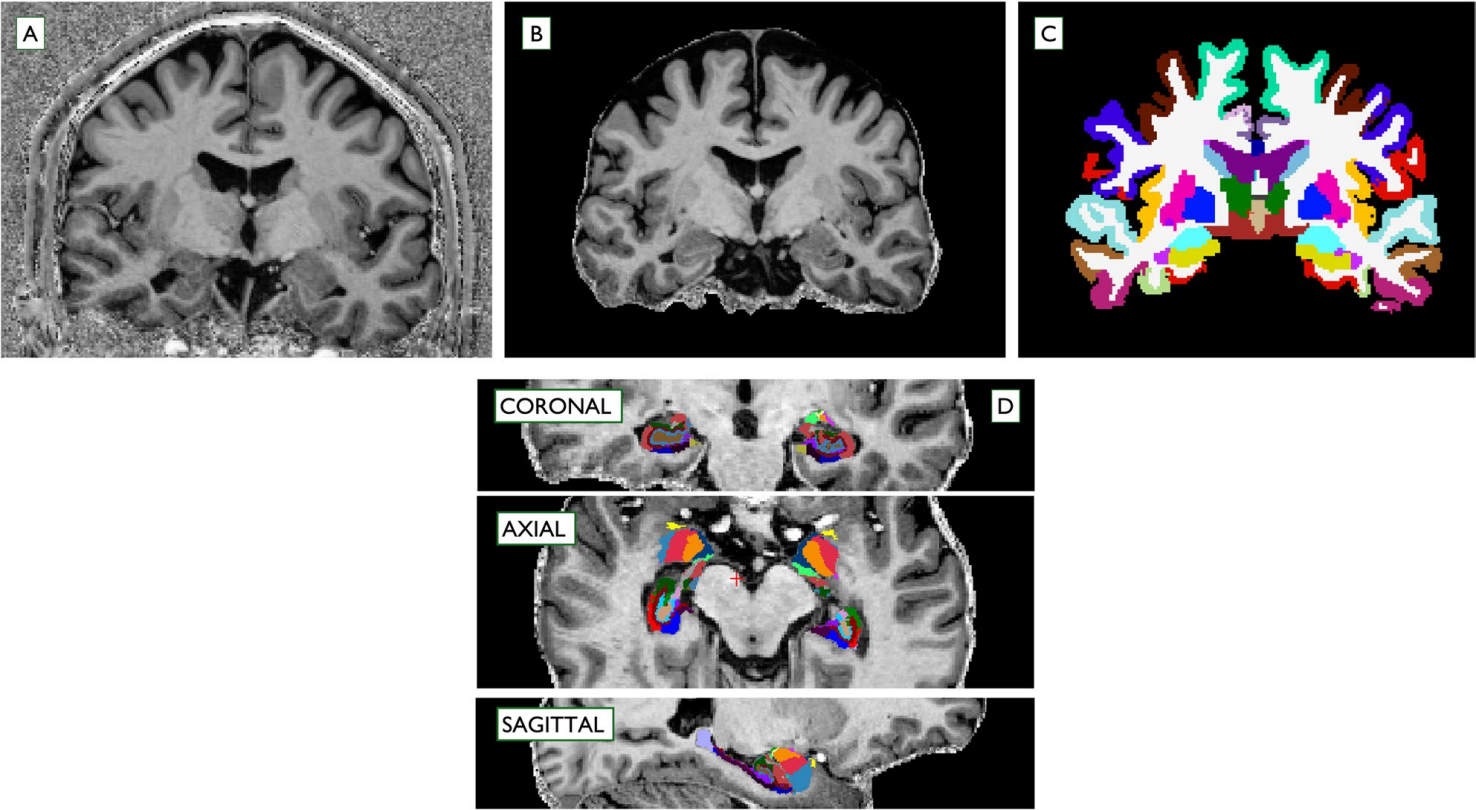
The structural preprocessing and subfield segmentation pipelines. **A** An original MP2RAGE image. **B** After skull-stripping with SynthStrip. **C** After segmentation with recon-all. **D** An example of hippocampal and amygdala segmentation in coronal, axial and sagittal orientations

Scans were skull-stripped using SynthStrip [39] and pre-processed using recon-all [40] both in FreeSurfer (Version 7.4.1 [41]). This resulted in individual volumes probabilistically segmented into regions of interest with an accuracy comparable to that of manual segmentation [40]. Within this automated pipeline, the Desikan-Killiany atlas is used for cortical parcellation [42], and the FreeSurfer Subcortical Probabilistic atlas is used for subcortical parcellation [43]. Failure occurred in 11 scans and was due to failure of white matter registration. Manual editing was minimal and only undertaken where the automated preprocessing and segmentation failed to complete. Editing was performed using recon-edit in FreeView (Version 3.0) in FreeSurfer by one researcher (EMcK) who was blind to diagnosis. Ten of these 11 scans were then successfully segmented using recon-all but one (subsequently found to belong to a control participant) failed a second time and was rejected from the analysis. Quality control of the scans was by visual inspection of the segmented images overlayed on the original T1 images in FreeView. Original images were checked for presence of incidental findings (such as space occupying lesions or tissue infarcts), and segmentation in the pre-processed volumes rated according to the degree of error in segmentation of white from grey matter. Of the 64 successfully processed scans, no scans were excluded due to incidental findings. Segmentation of cortical white and grey matter was rated as “good” or “adequate” in 57 cases; in the seven cases where cortical segmentation was rated as “poor” mis-registration of grey and white matter in peripheral areas (especially orbitofrontal, lateral and inferior temporal lobes, and cerebellum) was present. Loss of signal in peripheral brain areas is due to the increase in susceptibility effects and is a well-documented limitation of 7T imaging [44], in addition the relatively high failure rate of the recon-all command may reflect non-trivial processing challenges when using automated pipelines with 7T data. The primary analyses of interest were of medial temporal lobe subfields; since segmentation in this area was rated as “good” in all cases, all 64 scans were included in subsequent analyses. Region of interest volumes (subcortical), cortical thickness measures and total intracranial volume (TIV), were extracted for each individual as part of the recon-all pre-processing step. Medial temporal lobe subfield segmentation on the recon-all pre-processed data using the hippocampal and amygdala subfield tools in FreeSurfer [45, 46] (a complete list of the subfield volumes extracted are listed in the Supplementary materials). Quality control was again by visual inspection of the segmented images overlayed on the original T1 images in FreeView; all data passed QC. Images from the pipeline for cortical and subcortical segmentation and segmentation of subfields in Fig. 1. Further QC explanation and examples are shown in the Supplementary Materials.

### Statistical analysis

Data was visualised, statistical tests performed and figures generated using Jeffreys’s Amazing Statistics Program (JASP) [47] and R [48] in R studio [49] (references for individual packages can be found within the text). Normality of data was checked using Q-Q plots of residuals, and homogeneity of variances using Levene’s test. Statistical significance was set at *P* < 0.05 unless otherwise specified. Where appropriate, correction for multiple comparisons was applied (as described below) and both uncorrected and corrected values reported.

#### Missing data

Missing data for the visual hallucinations scale for the cohort was minor (6.7%). Missing data was visualised and explored using the Elegant Graphics for Data Analysis (ggplot2) package [50] in R. As per this exploration, data was deemed to be missing at random, and was, therefore, imputed using the Multivariate Imputation by Chained Equations (MICE) package [51] in R.

#### Statistical tests for group comparisons

Demographics (for example, age, sex and years of education), cognitive test scores and symptom questionnaire scores, were compared between patients and controls, and between diagnostic groups. Groups were compared using analysis of variance (ANOVA) with least squares difference (LSD) post-hoc tests for parametric data (e.g. age and plasma ptau-217), Kruskal-Wallis with Dunn post-hoc tests for non-parametric data (e.g. Addenbrookes Cognitive Examination scores), and Chi-squared tests for categorical data (e.g. sex). Post-hoc comparisons were corrected for a family of three using Holm correction.

For group comparisons of cortical thickness, region of interest and subfield volumes the R packages Companion to Applied Regression (car) [52] and Estimated Marginal Means (emmeans) [53] in R studio were used for the generation of generalised linear models (GLM) with Gaussian error structure. Age, sex, years of education, (and total intracranial volume (TIV) for regional volumetric comparisons), were included as covariates. The following model assumptions were tested using the car, emmeans, and ggplot2 R packages and found to be acceptable: approximate normality of residuals (Q-Q plots of model residuals), homoscedasticity (residuals vs. fitted values plots), absence of influential observations (Cook’s distance plots), adequate linearity of continuous covariates (partial residual plots), and absence of collinearity (variance inflation factors).

For all thickness and volumetric measures, measures from right and left hemispheres were combined in order to reduce the number of tests applied to the data because the DLB literature does not consistently suggest that atrophy is lateralised in this condition (e.g. see reviews [54, 55]). Type II ANOVAs were performed on the GLM models to determine the relative contributions of independent variables to thickness and volume variance. Where group was found to significantly influence variance in structural measures, post-hoc least squares difference (with Holm correction for family of three) were conducted to determine where group differences lay.

Extent of volumetric differences in hippocampal and amygdala subfields in AD compared to controls, and DLB compared to controls, was assessed by calculating subfield volumes as a proportion of individual TIV and comparing resulting means for patient with control groups. Non-parametric bootstrapping was used to obtain 95% confidence intervals. 2,000 resamples were selected as 95% confidence intervals were found to converge after approximately 1,500 resamples. Bootstrapping was by resampling with replacement using the Bootstrap R package (boot) [56] in R. Bias-corrected bootstrapping was not performed as the mathematical assumptions were not met in our dataset due to the modest sample size and small volumes of many subfields.

#### Statistical tests for associations

All correlations were calculated in R using the stats [48], tidyverse [57], and broom [58] packages. Correlations between residuals of a GLM were calculated (as described above but with diagnostic group omitted) and cognitive and symptom scores using Kendall’s correlations (suitable for non-parametric, ordinal data, and small data sets). *P* values were calculated with and without false discovery rate (FDR) multiple comparisons correction (correcting for the number of regions included in each segmentation, i.e. hippocampal subfields and amygdala subfield were corrected separately). Correlation scatterplots were created in JASP.

In addition, we explored associations between subfield volumes and cognitive and clinical data which are listed below. Particularly in the case of the subgroup analyses (exploring visual hallucinations and plasma ptau-217 concentrations) these analyses were based on small and uneven samples and were intended to be exploratory and hypothesis generating.

- In DLB and AD, associations between hippocampal subfield volumes and Addenbrookes Cognitive Examination scores.
- In DLB and AD, associations between amygdala subfield volumes and Addenbrookes Cognitive Examination scores.
- In the DLB group alone, (as no participants in the AD group reported visual hallucinations), associations between amygdala nuclei volumes and scores from the visual hallucinations rating scale.
- For the subgroup of participants with blood results, associations between subfield volumes and plasma ptau-217 concentrations.

## Results

The automated FreeSurfer pipeline was completed successfully in 64 of 65 participants. The cohort is described in Table 1 (with additional results in Supplementary Table 1). Groups were well-matched for age. Sex was unevenly distributed between groups, but this difference was not statistically significant. Education differed significantly between patients and controls but not between patient groups.

Plasma ptau-217 concentrations were available for exploratory analysis in a subset of 41 participants. Concentrations were significantly higher in the AD group with all participants reaching the suggested lower cut-off of 0.4 gl/ml [38] and lowest in controls, with concentrations in the DLB group occupying the intermediate space.

**Table 1:** Group comparisons of demographics, cognitive test scores and plasma ptau-217

	AD	DLB	HC	Group difference
*n*	25	20	19	
Age^a^	74.1 (8.1)	75.7 (7.1)	73.3 (5.7)	0.57
Sex^b^	16 M/ 9 F	15 M/ 5 F	10 M/ 9 F	2.12
Education^c^	13.6 (3.2)	13.6 (3.1)	17.2 (3.6)	12.47*^d, e^
ACE^c^	66.6 (10.2)	75.8 (11.7)	96.4 (3.4)	42.30*^d, e,f^
subgroup of participants with plasma ptau-217 results				
n	17	13	11	
Age^a^	71.8 (7.9)	76.4 (8.0)	72.3 (5.7)	1.58
Sex^b^	9 M/ 8 F	9 M/ 2 F	11 M/ 2 F	4.50
Education^c^	13.4 (2.8)	13.7 (3.4)	18.2 (3.5)	11.91*^d, e^
Plasma ptau217 concentration (pg/ml)^a^	1.09 (0.45)	0.72 (0.38)	0.27 (0.13)	17.25*^d, e,f^
Proportion with plasma ptau217 concentrations above 0.4 gl/ml	17	9	2	

Mean (standard deviation) by diagnostic group

Key: *ACE* Addenbrooke’s Cognitive Examination, *AD *Alzheimer’s disease, *DLB *dementia with Lewy bodies, *HC *healthy control

^a^Parametric data, F score for Type II ANOVA used to calculate group difference

^b^Categorical data, Chi-squared statistic used to calculate group difference

^c^Non-parametric data, Kruskal-Wallis statistic used to calculate group difference

^d^significant post hoc group difference AD-HC

^e^significant post hoc group difference DLB-HC

^f^significant post hoc group difference AD-DLB

*P < 0.05

### Group comparisons

#### Cortical thickness

Results of the GLM and subsequent Type 2 ANOVA and post-hoc tests for cortical thickness are summarised in Supplementary Tables 2 and represented in Supplementary Fig. 1. Mean percentage difference between groups is shown in Supplementary Table 3. Mean whole brain cortical thickness was smaller in patients than controls and smallest in AD with significant post-hoc differences between AD and controls, and DLB and controls. This pattern was repeated across much of the brain with most regions of the cortex thinner in AD than in other groups. Of interest, the following areas (which showed a significant overall group difference) were similar in thickness in AD and DLB: in the frontal lobe - pars opercularis, and rostral middle frontal cortex; in the parietal lobe - the superior parietal cortex; in the temporal lobe - the fusiform gyrus; in the occipital lobe - the cuneus, lateral occipital cortex, and lingual gyrus.

#### Subcortical brain volumes

Only the putamen and nucleus accumbens were significantly smaller in both DLB and AD compared to controls. Full results of the GLM and subsequent Type 2 ANOVA with least squares difference post-hoc tests (with Holm correction for family of three) are summarised in Supplementary Table 4. Mean percentage difference between groups is shown in Supplementary Table 5.

#### Medial temporal lobe subfield volumes

All hippocampal subfield volumes differed significantly between diagnostic groups except parasubiculum and hippocampal fissure. For all subfields, volumes were smallest in AD and largest in controls, with the DLB group sitting between the two. Four of the 18 subfields were significantly smaller in DLB compared to controls - molecular layer of the hippocampus proper, subiculum, presubiculum and entorhinal cortex. No subfields were significantly different between DLB and AD. Results of the GLM and subsequent Type 2 ANOVA and post-hoc tests are summarised in Supplementary Table 6. Raincloud plots showing group comparisons are found in Supplementary Fig. 2.

In DLB, largest mean volume differences compared to controls were in the entorhinal cortex (-22.5%; 95%CI: -12.6 to -31.6), subiculum (-21.5%; 95%CI: -11.9 to -30.6), presubiculum (-18.9%; 95%CI: -9.7 to − 27.7) and molecular layer of the hippocampus proper (-18.7%; 95%CI: -9.9 to -27.1). In AD, largest mean volume differences compared to controls were in the subiculum (-26.6%; 95%CI: -19.2 to -33.3), hippocampal-amygdala transition area (-25.7%; 95%CI: -17.1 to -33.6), presubiculum (-24.9%; 95%CI: -17.6 to -31.4) and entorhinal cortex (-24.3%; 95%CI: -14.4 to -33.3). Mean percentage difference between groups is shown in Table 2.

Whole amygdala and subfield volumes were significantly different across groups except for the paralaminar nucleus. Volumes in all subfields were smallest in AD and largest in controls and post-hoc analyses showed that these differences were statistically significant for both AD and DLB compared to controls in the accessory basal, central, medial, cortical nuclei and anterior amygdaloid area. There were no significant differences between AD and DLB. Results of the GLM and subsequent Type 2 ANOVA and post-hoc tests are summarised in Supplementary Table 6. Raincloud plots showing group comparisons are shown in Supplementary Fig. 2.

In the DLB group, largest mean volume differences were in the medial nucleus (-25.8%; 95%CI: -14.7 to -35.8), central nucleus (-24.7%; 95%CI: -14.5 to -34.0), cortical nucleus − 21.7%; 95%CI: -12.1 to -30.8) and accessory basal nucleus (-19.7%; 95%CI: -10.4 to -28.4). In the AD group, largest mean volume differences compared to controls were in the central nucleus (-26.9%; 95%CI: -19.8 to -33.4), medial nucleus (-26.8%; 95%CI: -18.5 to -35.1), cortical nucleus (-26.3%; 95%CI: -19.3 to -32.9) and accessory basal nucleus (-25.5%; 95%CI: -18.6 to -31.7). Mean percentage difference between groups are shown in Table 2.

**Table 2:** Mean % difference of subfield volumes (as proportion of TIV) (left and right volumes combined)

	AD % difference (vs. HC)	DLB % difference (vs. HC)	AD % difference (vs. DLB)
Hippocampus and subfields			
Whole hippocampus	-22.7* [-15.8 to -28.7]	-17.3 [-8.7 to -25.1]	-6.5 [3.0 to -14.5]
CA1	-19.7* [-12.5 to -26.4]	-16.3 [-7.7 to -24.3]	-4.0 [5.9 to -11.4]
CA2/3	-22.9* [-14.4 to -30.1]	-16.6 [-7.2 to -24.6]	-7.6 [2.0 to -16.3]
CA4	-21.3* [-14.2 to -27.7]	-16.4 [-8.1 to -23.5]	-5.8 [3.4 to -14.0]
MLHP	-24.2* [-17.6 to -30.5]	-18.7* [-9.9 to -27.1]	-6.8 [3.1 to -15.4]
GCMLDG	-21.4* [-14.1 to -27.7]	-16.2 [-7.5 to -24.0]	-6.1 [3.1 to -14.8]
Subiculum	-26.6* [-19.2 to -33.3]	-21.5* [-11.9 to -30.6]	-6.5 [5.2 to -16.7]
Presubiculum	-24.9* [-17.6 to -31.4]	-18.9* [-9.7 to -27.7]	-7.4 [3.8 to -16.3]
Parasubiculum	-14.1 [-0.1 to -25.8]	-11.0 [-0.5 to -20.9]	-3.4 [12.3 to -16.6]
HATA	-25.7* [-17.1 to -33.6]	-18.0 [-7.3 to -27.7]	-9.4 [3.3 to -20.1]
Fimbria	-24.9 [-10.9 to -36.6]	-15.2 [0.8 to -29.0]	-11.5 [7.3 to -26.2]
Tail	-22.0* [-13.9 to -29.1]	-14.8 [-4.9 to -24.3]	-8.5 [2.3 to -17.2]
Fissure	-8.8 [0.7 to -17.7]	-12.2 [-1.4 to -22.1]	3.9 [17.2 to -7.0]
Entorhinal cortex	-24.3* [-14.4 to -33.3]	-22.5* [-12.6 to -31.6]	-2.4 [9.7 to -13.6]
Amygdala and subfields			
Whole amygdala	-19.8* [-14.0 to -25.3]	-16.3 [-8.4 to -23.3]	-4.1 [5.5 to -12.1]
Accessory Basal	-25.5* [-18.6 to -31.7]	-19.7* [-10.4 to -28.4]	-7.2 [3.8 to -16.8]
AAA	-19.3* [-12.4 to -25.5]	-16.8* [-9.1 to 23.9]	-3.0 [6.8 to -11.7]
Central	-26.9* [-19.8 to -33.4]	-24.7* [-14.5 to -34.0]	-2.9 [10.2 to -13.7]
Medial	-26.8* [-18.5 to -35.1]	-25.8* [-14.7 to -35.8]	-1.3 [12.4 to -12.6]
Cortical	-26.3* [-19.3 to -32.9]	-21.7* [-12.1 to -30.8]	-5.9 [6.3 to -16.3]
CATA	-17.9* [-11.0 to -24.3]	-12.5 [-3.4 to -20.4]	-6.3 [3.6 to -14.2]
Paralaminar	-12.3 [-6.2 to -18.1]	-13.4 [-6.0 to -28.9]	1.3 [12.0 to -7.4]
Lateral	-18.0* [-11.5 to -23.5]	-14.9 [-7.8 to -22.4]	-3.6 [6.1 to -11.5]
Basal	-19.2* [-13.0 to -24.5]	-16.5 [-8.8 to -23.9]	-3.2 [7.4 to -11.6]

*AD *Alzheimer’s disease, *DLB *dementia with Lewy bodies, *HC *healthy control, *CA* cornu Ammonis, *MLHP* molecular layer hippocampus proper, *GCMLDG* granule cells of molecular layer and dentate gyrus, *HATA* hippocampal-amygdala transition area, *AAA* anterior amygdaloid area, *CATA* cortico-amygdala transition area

[ ] denote 95% confidence intervals

*P < 0.05 for post-hoc group comparison results from type 2 ANOVAs

### Associations

#### Associations with cognitive test scores

**Fig. 2:**
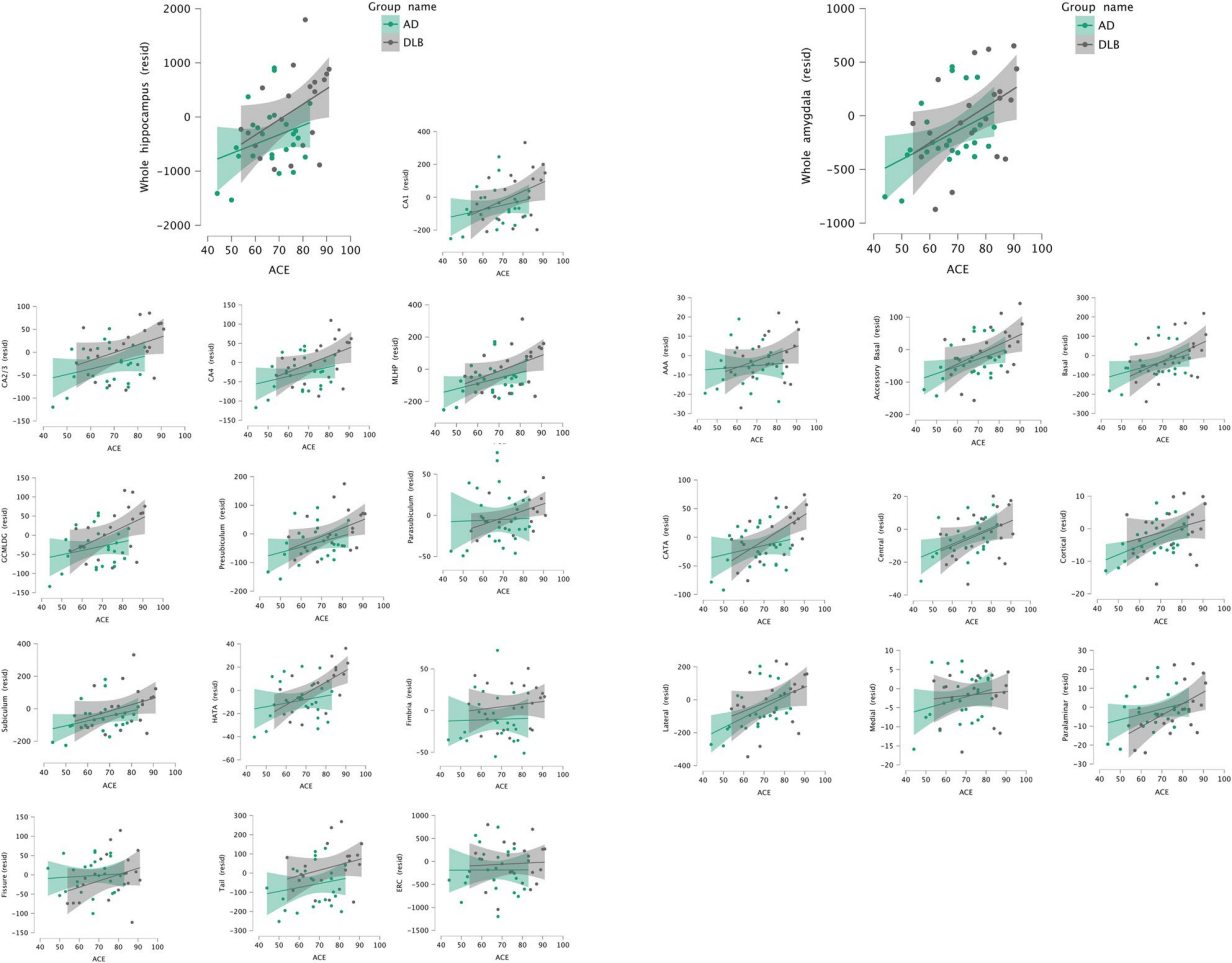
Scatterplots for hippocampal, amygdala and subfield volumes with Addenbrookes Cognitive Examination (ACE) scores. Data is for the combined patient group, left and right volumes are combined. Plotted values are residuals adjusted for age, sex, education and total intracranial volume. All correlations for the DLB group are significant *P* < 0.05 with false discovery rate correction for multiple comparisons except parasubiculum, fimbria, fissure, anterior-amygdaloid area (AAA) and medial subfield. Abbreviated subfields are listed in full in the main body of the text

In DLB, larger whole hippocampal and CA1, CA2/3, CA4, molecular layer of the hippocampus proper, granular layer of the dentate gyrus, hippocampal amygdala transition area and presubiculum volumes were significantly associated with higher total Addenbrookes Cognitive Examination-III scores (after correction for multiple comparisons with false discovery rate (FDR)). In DLB, larger whole amygdala and all amygdala subfield volumes (except anterior-amygdaloid area and medial nucleus) were significantly associated with higher total Addenbrookes Cognitive Examination-III scores (after correction for multiple comparisons with FDR). The full results are in Table 3 and scatterplots are in Fig. 2.

**Table 3:** Correlations for whole hippocampal, whole amygdala and respective subfield volumes (left and right volumes combined) with total Addenbrookes Cognitive Examination score (all patients, DLB, and AD)

	Kendall’s tau		
	All patients	DLB	AD
Whole hippocampus	0.382*^a^	0.442*^a^	0.113
Hippocampal subfields			
CA1	0.301*^a^	0.368*^a^	0.093
CA2/3	0.279*^a^	0.400*^a^	0.067
CA4	0.313*^a^	0.411 *^a^	0.073
MLHP	0.388*^a^	0.432*^a^	0.180
GCMLDG	0.305*^a^	0.474*^a^	0.027
HATA	0.354*^a^	0.600*^a^	0.087
Parasubiculum	0.210*	0.263	0.033
Presubiculum	0.406*^a^	0.442*^a^	0.127
Subiculum	0.392*^a^	0.305	0.160
Fimbria	0.135	0.137	− 0.040
Entorhinal cortex	0.107	0.200	− 0.060
Hippocampal tail	0.382*^a^	0.189	0.027
Hippocampal fissure	0.160	0.253	0.047
Whole amygdala	0.414*^a^	0.442*^a^	0.300
Amygdala subfields			
Lateral nucleus	0.376*^a^	0.337*	0.373
Basal nucleus	0.376*^a^	0.442*^a^	0.193
Accessory Basal nucleus	0.398*^a^	0.526*^a^	0.267
AAA	0.194	0.295	0.067
Central nucleus	0.307*^a^	0.547*^a^	0.233
Medial nucleus	0.170	0.137	0.067
Cortical nucleus	0.352*^a^	0.368*^a^	0.267
CATA	0.333*^a^	0.442*^a^	0.073
Paralaminar nucleus	0.285*^a^	0.326*	0.053

Plotted values are residuals adjusted for age, sex, education and total intracranial volume

*CA* cornu Ammonis, *MLHP* molecular layer hippocampus proper, *GCMLDG* granule cells of molecular layer and dentate gyrus, *HATA* hippocampal-amygdala transition area, *AAA* anterior amygdaloid area, *CATA* cortico-amygdala transition area

*P < 0.05

^a^survives correction for multiple comparisons with false discovery rate (FDR) – FDR computed separately for hippocampal and amygdala

#### Associations with visual hallucination questionnaire scores (exploratory)

In the DLB group alone, only the association between smaller cortico-amygdaloid transition area volumes and higher visual hallucinations questionnaire part 2 scores (i.e. with greater frequency and duration of visual hallucinations) survived correction for multiple comparisons. In additional exploratory analyses where ACE-III scores were added as an additional covariate to correct for disease severity, no associations were significant (Supplementary Table 7). In the DLB group alone, no associations between smaller whole amygdala and amygdala subfield volumes and higher visual hallucinations questionnaire part 3 scores (i.e. with stronger emotional responses to visual hallucinations) survived correction for multiple comparisons. In additional exploratory analyses where ACE-III scores were added as an additional covariate to correct for disease severity, no associations survived correction for multiple comparisons (Supplementary Table 7). Results are in Table 4 and scatter plots for whole amygdala are in Supplementary Fig. 3.

**Table 4:** Correlations for amygdala and subfield volumes (left and right volumes combined) with visual hallucinations part 2 (frequency and duration) and part 3 (emotional response) scores (dementia with Lewy bodies group only)

	VH part 2 scores	VH part 3 scores
	Kendall’s tau	
Whole amygdala	-0.326*	-0.392*
Subfields		
Lateral nucleus	-0.179	-0.335*
Basal nucleus	-0.326*	-0.324
Accessory Basal nucleus	-0.411*	-0.347*
AAA	-0.347*	-0.449*
Central nucleus	-0.242	-0.313
Medial nucleus	-0.063	-0.051
Cortical nucleus	-0.316	-0.358*
CATA	-0.453*^a^	-0.313
Paralaminar nucleus	-0.189	-0.313

Plotted values are residuals adjusted for age, sex, education and total intracranial volume

*AAA* anterior amygdaloid area, *CATA* cortico-amygdala transition area, *VH *visual hallucinations

*P < 0.05

^a^survives correction for multiple comparisons with false discovery rate

#### Associations with plasma ptau-217 concentrations (exploratory)

**Fig. 3:**
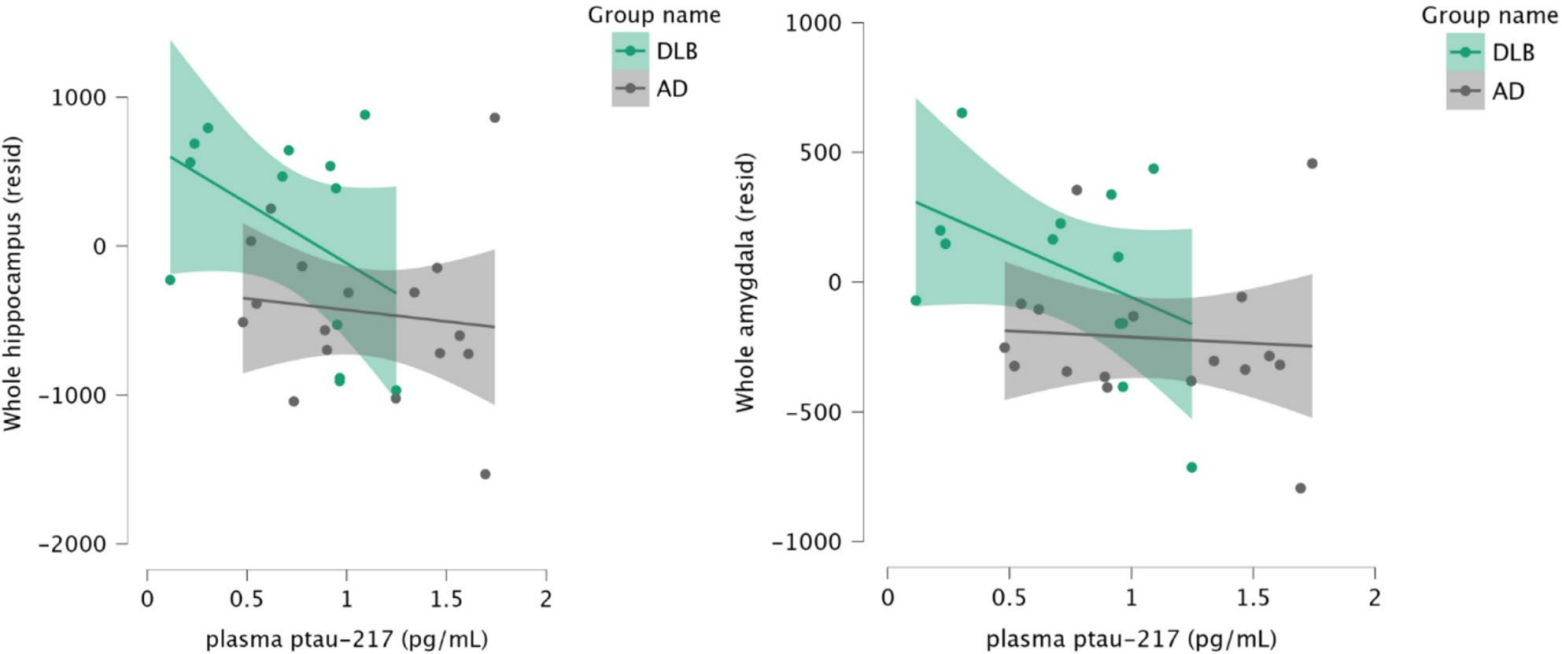
Scatter plots for whole hippocampal and amygdala volumes with plasma ptau-217 concentrations. Data is for all patients in the exploratory bloods subgroup, left and right volumes are combined. Plotted values are residuals (resid) adjusted for age, sex, education, and total intracranial volume. Correlations are significant *P*_FDR_<0.05

In the combined patient group within the exploratory bloods subgroup, higher plasma ptau-217 concentrations were significantly associated with smaller volumes of whole hippocampus, CA2/3, CA4, molecular layer of the hippocampus proper, granular cells of the molecular layer and dentate gyrus, hippocampal-amygdala transition area, presubiculum, subiculum, and hippocampal tail, whole amygdala, lateral, basal, accessory basal, central, and cortical amygdala nuclei after correction for multiple comparisons (with FDR). Full results in Table 5. Scatter plots for whole hippocampus and whole amygdala in Fig. 3. In addition, comparison of whole hippocampal and whole amygdala volumes in patients categorised according to diagnosis and presence or absence of raised plasma ptau-217 concentrations (using cut-offs suggested in [38]) are shown in Supplementary Tables 8 and Supplementary Fig. 5.

**Table 5:** Correlations for hippocampal, amygdala and subfield volumes(left and right volumes combined) with plasma ptau-217 concentrations (all patients in the bloods subgroup, *n* = 40)

	All patients in ptau-217 subgroup		
	Kendall’s tau		Kendall’s tau
Whole hippocampus	-0.263*^a^	Whole amygdala	-0.251*^a^
Hippocampal subfields		Amygdala subfields	
CA1	-0.154	Lateral nucleus	-0.249*^a^
CA2/3	-0.268*^a^	Basal nucleus	-0.227*
CA4	-0.241*^a^	Accessory Basal nucleus	-0.337*^a^
MLHP	-0.249*^a^	AAA	-0.180
GCMLDG	-0.244*^a^	Central nucleus	-0.251*^a^
HATA	-0.246*^a^	Medial nucleus	-0.144
Parasubiculum	-0.0561	Cortical nucleus	-0.295*^a^
Presubiculum	-0.298*^a^	CATA	-0.146
Subiculum	-0.254*^a^	Paralaminar nucleus	-0.0707
Fimbria	-0.124		
Entorhinal cortex	-0.0488		
Hippocampal tail	-0.266*^a^		
Hippocampal fissure	-0.0463		

Plotted values are residuals adjusted for age, sex, education and total intracranial volume

*CA *cornu Ammonis, *MLHP* molecular layer hippocampus proper, *GCMLDG *granular cells of the molecular layer and dentate gyrus, *AAA* anterior amygdaloid area, *CATA *cortico-amygdala transition area

*P < 0.05

^a^survives correction for multiple comparisons with false discovery rate (hippocampal and amygdala fields corrected separately)

## Discussion

### Cortical thickness and volumetric analyses

Our cortical thickness and subcortical volume analyses revealed few regions where DLB and AD were significantly different. A similar pattern of cortical thinning in DLB and AD (with less marked thinning in DLB than AD is in keeping with the previous literature [4]. Though subcortical volumes were smaller in DLB than in controls, the lack of significant differences was perhaps surprising [6]. Overall, neither whole brain measures, nor subfield volumes differentiated well between the patient groups. It may be that the study was not adequately powered to detect these differences, as sample size calculations were powered to detect hippocampal subfield volume differences between DLB and controls and were informed by data from 7T studies of AD vs. controls (due to a lack of studies in DLB at 7T and a lack of studies directly comparing subfields between AD and DLB cohorts). However, the absence of clear differentiation between the DLB and AD groups in this modest cohort is itself of interest, as it suggests that systematic structural differences between DLB and AD may be small and not be apparent at the individual level, an important consideration from a clinical perspective. Another possibility is that in this clinically-defined cohort, there was a substantial degree of AD-co-pathology, which is well-recognised to occur in DLB [59].

### Hippocampal subfield atrophy

Our primary analysis of interest was the comparison of subfields of medial temporal lobe structures between DLB and controls. In DLB, hippocampal subfield volumes were reduced by around 10%-20% compared to controls; there are no previous 7T hippocampal subfield studies in DLB, findings at 3T have suggested that subfields are reduced by around 2%-5% to 10%-15% compared to controls [15, 16]. In the AD group, hippocampal subfield volumes were reduced by around 10%-30% compared to controls, in keeping with previous findings in AD at 7T [34]; at 3T previous findings have been comparable [16] or a little less pronounced [15]. Overall, these findings are consistent with the previous literature which has found that the medial temporal lobe is relatively less atrophied in DLB compared to AD [3, 9], but also suggests that, compared to controls, significant atrophy is found across hippocampal subfields in DLB. A greater degree of hippocampal subfield atrophy in the DLB group than previously found may be due to the of increase in MRI field strength, i.e. with higher tissue contrast resolution, hippocampal subfield atrophy in DLB, which is more subtle than that seen in AD, are revealed. However, since no clear increase was seen in the AD group, there may be other explanations for the difference in our cohort, for example, the impact of AD-co-pathology in the DLB group.

Direct comparison with previous studies is complicated by the use of a variety of segmentation techniques, both manual and automated [32]. Subfields and layers which are difficult to differentiate on MRI may be considered together, such as the cornu Ammonis 2/3 subfield in the segmentation pipeline used in this analysis, or the often-used CA1-SRLM which combines the stratum radiatum and stratum lacunosum-moleculare of the cornu Ammonis (CA) 1 [60]. The increasing use of automated segmentation pipelines (which are less labour intensive, and have higher inter-rater reliability [61]) may contribute to more consistent methodologies.

We hypothesised that CA2/3 and the entorhinal cortex would be significantly smaller in DLB compared to controls, as these are the medial temporal lobe areas most severely affected by Lewy body pathology at postmortem [62]. Although CA2/ 3 subfield volumes were smaller in DLB than controls, the difference was not statistically significant. The entorhinal cortex was significantly smaller in both AD and DLB compared to controls, and mean volumes and spread looked very similar in the AD and DLB groups. In AD the entorhinal cortex is identified as an area of early involvement; tau accumulates in the entorhinal cortex before spreading to the hippocampus and cortex [63], most likely in a transsynaptic manner [64]. Early, associated atrophy of the entorhinal cortex in AD is also noted and appears to be due to early and profound loss of layer 2 neurones [65]. In DLB, Lewy body pathology colocalises with tau pathology in the entorhinal cortex [66] and entorhinal cortex pathology may propagate to CA2/3 directly; although a direct link between the entorhinal cortex and CA2 subfield has not been confirmed in humans, it has been described in rodents and non-human primates [67].

In this analysis, the entorhinal cortex was segmented in its entirety, however, it is itself a relatively large structure (around half the volume of the whole hippocampus). It is, itself a complex structure which can be divided according to its cytoarchitecture and has been segmented into eight distinct regions [68], though, with the exception of the olfactory subfield, the individual functions of these regions have not yet been described [69]. On MRI these eight subfields have been successfully segmented using postmortem 7T MRI [69]; in vivo imaging is currently restricted by very long scan times but may be an interesting future development.

### Amygdala subfield atrophy

In DLB, accessory basal, central, medial and cortical nuclei, and anterior amygdaloid area volumes were reduced by around 16%-26%. compared to controls; in these same nuclei reductions in the AD group were similar (around 19%-27%). The degree of volumetric subfield difference in both AD and DLB was more pronounced in the amygdala than in the hippocampus. This suggests that in mild to moderate DLB, amygdala changes are more marked than hippocampal changes, and that in mild to moderate AD amygdala subfield atrophy is as severe (or more severe) than is seen in hippocampal subfields.

Our amygdala findings in the AD group are echoed by the only study of the amygdala at 7T in AD which found amygdala atrophy across all measured subfields, with largest effects in central, medial, and cortical nuclei [34], and more pronounced amygdala than hippocampal subfield atrophy across the AD continuum. Our findings in DLB are also consistent with the literature, in neuropathological studies the central and accessory basal nucleus are identified as those most significantly affected in DLB [70]. The central nucleus has been identified as playing a role in the consolidation of fearful memories [71] and the accessory basal nucleus (alongside the basal nucleus) is the main input area to the ventral striatum [72], an area responsible for the integration of knowledge about reward expectation with behaviour [73]. The cortical and medial nuclei (which were also found to be significantly smaller in DLB compared to controls) are involved in olfaction and in the regulation of social behaviour [74].

### Biomarkers and Alzheimer’s co-pathology

The cohort was defined clinically without biomarker confirmation, meaning that the inclusion of participants with pathologies such as Limbic-predominant age-related TDP-43 encephalopathy neuropathologic change (LATE-NC, which may present in a similar clinical fashion to AD) cannot be ruled-out. The lack of biomarker confirmation is a limitation in the study, and means that the lack of differentiation between patient groups could potentially be due to misclassification of participants or a greater than usual degree of AD co-pathology in our cohort. In a pathological DLB cohort, antemortem MRI entorhinal cortex volume loss was greater in those with Alzheimer’s co- pathology at postmortem and was associated with concomitant entorhinal tau [66].

An exploratory analysis was performed in a subset of participants using plasma ptau-217 concentrations for the measurement of Alzheimer’s co-pathology [38], and the results tentatively support our diagnostic classification, with 7/17 (100%) participants in the AD subgroup and 9/13 (69%) of participants in the DLB subgroup showing evidence of amyloid-related pathology. For the DLB group this is in keeping with the literature [75]. However, although very promising, plasma ptau-217 is yet routinely used as a clinical diagnostic biomarker [76] and its significance in DLB is not yet fully defined [77]. The development of a similar blood-based biomarker for alpha-synuclein pathology is ongoing [78].

The exploratory plasma ptau-217 subgroup analysis showing strong associations between medial temporal lobe subfield volumes and plasma ptau-217 concentrations is limited due to the small and uneven sample size; reproduction of these findings in a larger, adequately powered sample could support the theory that decreases in hippocampal subfield volumes in DLB are driven by Alzheimer’s rather than “pure” Lewy body pathology.

### Association analyses

In DLB, significant positive correlations between Addenbrookes Cognitive Examination scores and hippocampal and amygdala subfield volumes builds on previous research in which scores of global cognition and memory function have been found to positively correlate with cornu Ammonis 1 subfield volumes in AD [32], and in Parkinson’s disease dementia/ Parkinson’s disease with mild cognitive impairment [79]. Intriguingly, when separated by diagnosis, correlations between hippocampal subfield volumes and total Addenbrookes Cognitive Examination scores are highly significant in the DLB group and non-significant in the AD group. One explanation for this is that hippocampal atrophy is more closely linked to cognition in DLB where atrophy is less profound and may occur later than in AD [8]. These results may reflect global medial temporal lobe atrophy rather than specific subfield effects since the same pattern was seen across the hippocampus. The significant positive correlations between Addenbrookes Cognitive Examination scores and amygdala are also supported by previous findings at 7T, where volumes of amygdala subfields were found to correlate with global cognition [34]. The Addenbrookes Cognitive Examination is a non-specific measure of global cognition, these analyses would have been strengthened by the inclusion of more domain-specific measures such as those measuring episodic memory function or emotional processing speeds, unfortunately, this data was not collected in this cohort.

In DLB, an exploratory subgroup analysis of amygdala subfield volumes suggested that there may be a possible relationship between decreasing volumes in amygdala subfields and greater frequency and duration of visual hallucinations, and stronger emotional response to visual hallucinations. However, the analysis results were not found to be robust to the addition of covariates to control for severity of disease. Alterations in amygdala volumes and connectivity are implicated in the production of hallucinations in psychosis (though these are usually auditory in nature) [28], and the production of visual hallucinations on direct electrical stimulation of the cortical (also called superficial) nuclei [29] have also been described. The number of non-hallucinators in our DLB sample was extremely small; collection of data from a cohort with a larger number of non-hallucinators would be especially interesting but may be challenging as visual hallucinations are present in around 80% of clinical cases [30].

### Further strengths and limitations

This analysis of structural changes at 7T was conducted on a cohort of well-described, clinically defined, patients who met the current diagnostic criteria for DLB or AD at the time of recruitment. It was adequately powered to detect changes in hippocampal subfields in DLB at 7T, the primary outcome of the study. For a 7T cohort, the number of participants compares favourably with other similar studies [32]; it is challenging to recruit older adults for 7T imaging, safety procedures are necessarily more rigorous than at lower field strengths [80], and many potential participants were excluded from the study due to previous surgical and medical procedures.

This study highlights the specific challenges of 7T imaging which include the impact of additional safety-checks on recruitment, non-trivial processing challenges (as demonstrated by the relatively high failure rate of the initial preprocessing steps), and the current lack of validation of automated segmentation approaches at 7T field strength; freely accessible pipelines are currently validated for 3T (not 7T) images, though other groups have successfully used automated pipelines at 7T [34, 81]. Methods papers focusing on the comparison and validation of such pipelines at 7T and consensus papers outlining best practice in this emerging field would improve the reliability and reproducibility of 7T imaging studies.

A limitation of the cohort is the uneven sex distribution, with more males than females in both patient groups and an even split in controls. A predominance of males in the DLB group is not unexpected since postmortem confirmed Lewy pathology is around three times more common in males than females [82]. However, 61% of the Alzheimer’s group was male while around two thirds of those with AD are female [83]. One reason for the disparity in our cohort is that the additional restrictions on eligibility for 7T MRI may have meant that men were more likely to be accepted for scanning than women (for example, two common surgeries in those we screened for eligibility and excluded, were cholecystectomy and hysterectomy; surgeries more commonly/ exclusively performed in females). The uneven distribution of males and females in the cohort has implications for subsequent analyses and interpretation of findings, especially since sex differences may play a role in the aetiology and pathological evolution of AD and other dementias [84]. In addition, this cohort may not be representative of the general population. As evidenced by the significant difference in education between patients and controls, the control cohort is particularly highly educated. Adjustment for years of education in the analyses is intended to correct for this between group imbalance but some effects may persist. Further, due to rigorous scanning safety protocols, the study population may also be in better general health than is typical for patients and older people as many common medical and surgical procedures, such as pacemaker or cholecystectomy excluded potential participants from enrolling in the study.

Finally, the analyses presented are cross-sectional which limits conclusions regarding causality and disease progression. For example, properly considered, “atrophy” can only be determined, rather than inferred, if data is collected at multiple timepoints. Future work may focus on the collection of longitudinal data.

## Conclusions

In this prospective, cross-sectional, seven tesla imaging study (7T-DLB), using structural imaging and automated subfield segmentation pipelines we found hippocampal and amygdala subfield atrophy in DLB compared to controls, as well as in AD compared to controls, where it has previously been described. In additional analyses, we examined links between subfield volumes, cognitive and non-cognitive symptoms, and markers of amyloid pathology; although our analyses were exploratory and findings tentative, they stimulate discussion and provoke us to pose further questions about the neural correlates of DLB symptoms and the role that AD co-pathology may play in this disease.

## Supplementary Information

Supplementary Material 1.

## Data Availability

Data are available on reasonable request to the principal investigator.
