# Supplementary material for: Seven tesla MRI reveals amygdala and hippocampal subfield atrophy in dementia with Lewy bodies
Source: Alzheimers Res Ther. 2026 May 27;18:173. doi: 10.1186/s13195-026-02091-8 (PMC13404107; doi:10.1186/s13195-026-02091-8)
Supplement: Supplementary file 1 — Supplementary Material 1. [file 13195_2026_2091_MOESM1_ESM.pdf]

Supplementary materials for "Seven tesla MRI reveals significant amygdala and hippocampal subfield atrophy in dementia with Lewy bodies"

Supplementary Table 1 Additional group comparisons of demographics, cognitive test scores and plasma ptau-217

	**AD mean (SD)**	**DLB mean (SD)**	**HC mean (SD)**	**Group difference**
**n**	**25**	**20**	**19**	
**GDS[a]**	3.0 (1.9)	5.4 (4.1)	1.5 (2.6)	**15.93*[cd]**
**BADLS[a]**	9.7 (8.4)	11.1 (7.5)	NA	0.78
**UPDRS[a]**	5.2 (8.1)	28.1 (16.0)	2.6 (4.2)	**33.03*[de]**
**VH[b]**	0+/25-	17+/3-	NA	**58.93*[e]**
**VH score[a]**	0.0 (0.0)	10.6 (5.4)	0.0 (0.0)	**31.09*[de]**
**CF[b]**	9+/16-	12+/8-	NA	2.57
**CAF[a]**	3.5 (4.1)	5.6 (3.6)	NA	**3.40*[e]**
**RBD[††]**	4+/21-	13+/7-	NA	**11.35*[e]**
**RBD severity[†]**	.1 (.4)	1.1 (1.4)	NA	**14.20*[e]**

AD=Alzheimer's disease; DLB=dementia with Lewy bodies; HC=healthy control; SD=standard deviation; GDS= Geriatric Depression Scale (15 point version) [1]; BADLS= Bristol Activities of Daily Living Score; UPDRS= Unified Parkinson's Disease Rating Scale-part III (motor) [2]; VH= visual hallucinations (presence or absence on screening); VH score= visual hallucinations questionnaire part 2 score (frequency and duration); CAF= Clinical Assessment of fluctuations score; CF= cognitive fluctuations (presence or absence, defined as CAF score >5 [3]); RBD= positive or negative response to the REM-sleep behaviour disorder questionnaire screening question [4]; RBD severity= RBD questionnaire score

[a] Non-parametric data, Kruskal-Wallis statistic used to calculate group difference

[b]Categorical data, Chi-squared statistic used to calculate group difference

[c]significant post hoc group difference AD-HC

[d]significant post hoc group difference DLB-HC

[e]significant post hoc group difference AD-DLB

*P < 0.05

Supplementary Table 2 Group comparisons of regional mean cortical thickness

		AD mean (SD) n=25	DLB mean (SD) n=20	HC mean (SD) n=19	Group difference	Effect size η_p^2
	**Whole brain**	2.14 (0.08)	2.16 (0.08)	2.25 (0.07)	**11.88*[ab]**	**.29**
Frontal	**Caudal middle frontal**	2.22 (0.13)	2.27 (0.14)	2.37 (0.10)	**4.77*[ab]**	**.14**
Frontal	**Frontal pole**	2.49 (0.17)	2.43 (0.14)	2.55 (0.19)	**5.72***	**.16**
Frontal	**Lateral orbitofrontal**	2.25 (0.10)	2.24 (0.11)	2.28 (0.09)	0.94	.03
Frontal	**Medial orbitofrontal**	2.18 (0.15)	2.22 (0.18)	2.15 (0.11)	0.54	.02
Frontal	**Pars opercularis**	2.26 (0.11)	2.28 (0.10)	2.37 (0.10)	**5.00*[ab]**	**.15**
Frontal	**Pars orbitalis**	2.29 (0.13)	2.26 (0.15)	2.34 (0.13)	0.92	.03
Frontal	**Pars triangularis**	2.20 (0.10)	2.19 (0.11)	2.27 (0.09)	**3.42*[ab]**	**.11**
Frontal	**Precentral**	2.35 (0.14)	2.28 (0.17)	2.41 (0.14)	2.53	.08
Frontal	**Rostral middle frontal**	2.10 (0.12)	2.11 (0.12)	2.22 (0.11)	**6.35*[ab]**	**.18**
Frontal	**Rostral anterior cingulate**	2.54 (0.17)	2.57 (0.17)	2.50 (0.16)	0.13	.00
Frontal	**Caudal anterior cingulate**	2.30 (0.18)	2.23 (0.20)	2.29 (0.18)	1.38	.05
Frontal	**Superior frontal**	2.38 (0.12)	2.40 (0.13)	2.50 (0.10)	**4.97*[ab]**	**.15**
Parietal	**Inferior parietal**	2.07 (0.14)	2.15 (0.10)	2.26 (0.09)	**18.50*[abc]**	**.39**
Parietal	**Paracentral**	2.22 (0.13)	2.19 (0.13)	2.24 (0.10)	1.38	.05
Parietal	**Postcentral**	1.93 (0.10)	1.89 (0.10)	1.96 (0.09)	2.45	.08
Parietal	**Precuneus**	2.01 (0.14)	2.07 (0.12)	2.17 (0.09)	**12.07*[ab]**	**.29**
Parietal	**Superior parietal**	1.94 (0.14)	1.95 (0.13)	2.03 (0.11)	**4.13***	**.12**
Parietal	**Supramarginal**	2.17 (0.11)	2.23 (0.10)	2.32 (0.09)	**12.29*[ab]**	**.30**
Temporal	**Banks superior temporal sulcus**	2.07 (0.14)	2.16 (0.11)	2.26 (0.11)	**13.10*[abc]**	**.31**
Temporal	**Entorhinal cortex**	2.27 (0.27)	2.39 (0.22)	2.66 (0.21)	**14.86*[ab]**	**.34**
Temporal	**Fusiform gyrus**	2.18 (0.12)	2.17 (0.08)	2.29 (0.11)	**7.58*[ab]**	**.21**
Temporal	**Inferior temporal**	2.14 (0.11)	2.17 (0.10)	2.30 (0.11)	**12.47*[ab]**	**.30**
Temporal	**Middle temporal**	2.23 (0.13)	2.33 (0.12)	2.40 (0.13)	**12.35*[ac]**	**.30**
Temporal	**Parahippocampus**	2.17 (0.23)	2.07 (0.19)	2.32 (0.20)	**6.41*[ab]**	**.18**
Temporal	**Superior temporal**	2.27 (0.16)	2.33 (0.13)	2.46 (0.15)	**7.92*[ab]**	**.21**
Temporal	**Temporal pole**	3.02 (0.29)	3.01 (0.25)	3.24 (0.32)	**3.48*[ab]**	**.11**
Temporal	**Transverse temporal**	2.12 (0.15)	2.06 (0.16)	2.17 (0.22)	1.40	.05
Occipital	**Cuneus**	1.73 (0.11)	1.72 (0.10)	1.77 (0.08)	**4.27***	**.13**
Occipital	**Lateral occipital cortex**	1.97 (0.11)	1.96 (0.13)	2.08 (0.10)	**8.24*[ab]**	**.22**
Occipital	**Lingual**	1.82 (0.09)	1.83 (0.09)	1.91 (0.11)	**8.13*[ab]**	**.22**
Occipital	**Pericalcarine**	1.56 (0.09)	1.55 (0.09)	1.59 (0.07)	1.23	.04
	**Insula**	2.59 (0.15)	2.65 (0.16)	2.68 (0.15)	**3.18***	**.10**

Group comparisons of regional mean cortical thickness in mm (left and right averaged). Results of the GLM (adjusting for age, sex and education) with subsequent Type 2 ANOVA and least squares difference post-hoc tests (with Holm correction for family of three).
AD=Alzheimer's disease; DLB=dementia with Lewy bodies; HC=healthy control; SD=standard deviation

[a]significant post hoc group difference AD-HC

[b]significant post hoc group difference DLB-HC

[c]significant post hoc group difference AD-DLB

*$P < 0.05$

Supplementary Table 3 Mean % difference in cortical thickness

	Region	AD % difference (vs HC)	DLB % difference (vs HC)	AD % difference (vs DLB)
Frontal	Caudal middle frontal	**-6.2* [-3.3 to -9.0]**	**-4.3* [-1.2 to -7.4]**	-2.0 [1.4 to -5.5]
	Frontal pole	-2.4 [2.0 to -6.2]	-4.8 [-1.0 to -8.7]	2.5 [6.5 to -1.0]
	Lateral orbitofrontal	-1.3 [1.2 to -3.8]	-1.6 [1.1 to -4.4]	0.2 [3.0 to -2.4]
	Medial orbitofrontal	1.5 [5.2 to -2.0]	3.4 [7.8 to -0.7]	-1.9 [2.6 to -6.1]
	Pars opercularis	**-4.5* [-1.7 to -6.9]**	**-3.8* [-1.0 to -6.2]**	-0.7 [2.0 to -3.4]
	Pars orbitalis	-2.2 [1.1 to -5.5]	-3.5 [0.2 to -7.1]	1.4 [5.0 to -2.2]
	Pars triangularis	**-3.2* [-0.9 to -5.7]**	**-3.8* [-1.0 to -6.5]**	0.5 [3.3 to -2.5]
	Precentral	-2.4 [1.0 to -5.7]	-5.5 [-1.3 to -9.2]	3.3 [7.4 to -0.9]
	Rostral middle frontal	**-5.6* [-2.6 to -9.4]**	**-5.1* [-1.9 to -8.1]**	-0.6 [2.7 to -3.7]
	Rostral anterior cingulate	1.8 [5.7 to -2.1]	2.7 [7.3 to -1.5]	-0.9 [2.9 to -4.8]
	Caudal anterior cingulate	0.2 [5.1 to -4.2]	-2.8 [2.7 to -7.8]	3.1 [8.5 to -2.0]
	Superior frontal	**-4.8* [-2.2 to -7.3]**	**-3.9* [-1.1 to -6.8]**	-1.0 [2.3 to -4.1]
Parietal	Inferior parietal	**-8.6* [-5.8 to -11.7]**	**-4.8* [-2.3 to -7.4]**	**-4.0* [-0.8 to -7.0]**
	Paracentral	-0.8 [2.1 to -3.8]	-2.1 [0.9 to -5.3]	1.3 [4.8 to -2.1]
	Postcentral	-1.7 [1.2 to -4.5]	-3.4 [-0.5 to -6.2]	1.8 [4.9 to -1.3]
	Precuneus	**-7.3* [-4.3 to -10.2]**	**-4.7* [-1.8 to -7.7]**	-2.7 [1.0 to -6.4]
	Superior parietal	-4.5 [-0.7 to -8.2]	-3.9 [-0.3 to -7.5]	-0.6 [3.7 to -4.5]
	Supramarginal	**-6.4* [-4.0 to -8.9]**	**-4.0* [-1.3 to -6.6]**	-2.5 [0.3 to -5.1]
Temporal	Banks superior temporal sulcus	**-8.2* [-4.5 to -11.4]**	**-4.5* [-1.4 to -7.4]**	**-3.9* [-0.6 to -7.2]**
	Entorhinal cortex	**-14.4* [-9.6 to -19.2]**	**-10.1* [-5.1 to -14.7]**	-4.9 [1.0 to -10.6]
	Fusiform gyrus	**-4.8* [-1.9 to -7.7]**	**-5.2* [-2.6 to -7.7]**	0.4 [2.9 to -2.3]
	Inferior temporal	**-6.9* [-3.9 to -9.7]**	**-5.6* [-2.8 to -8.2]**	-1.4 [1.4 to -4.3]
	Middle temporal	**-7.3* [-4.1 to -10.5]**	-3.2 [0.1 to -6.4]	**-4.3* [-1.2 to -7.2]**
	Parahippocampus	**-6.6* [-1.1 to -11.6]**	**-10.8* [-6.0 to -15.3]**	4.7 [10.9 to -1.1]
	Superior temporal	**-7.5* [-4.0 to -10.8]**	**-5.2* [-1.6 to -8.5]**	-2.4 [1.3 to -5.8]
	Temporal pole	**-6.6* [-1.0 to -11.8]**	**-7.1* [-1.6 to -12.3]**	0.5 [5.6 to -4.8]
	Transverse temporal	-2.4 [3.1 to -7.3]	-5.0 [0.6 to -10.1]	2.7 [7.1 to -1.6]
Occipital	Cuneus	-2.7 [0.5 to -6.0]	-3.1 [0.2 to -6.2]	0.5 [4.1 to -3.0]
	Lateral occipital cortex	**-5.2* [-2.5 to -8.1]**	**-5.5* [-2.2 to -8.9]**	0.4 [4.1 to -3.1]
	Lingual	**-4.9* [-1.8 to -8.0]**	**-4.3* [-0.7 to -7.4]**	-0.7 [1.9 to -3.7]
	Pericalcarine	-1.8 [1.2 to -4.8]	-2.2 [1.1 to -5.3]	0.4 [3.9 to -3.1]
	Insula	-3.2 [0.4 to -6.3]	-0.9 [2.8 to -4.3]	-2.3 [1.1 to -5.6]

Mean % difference in cortical thickness (left and right hemisphere values combined and averaged). Significance values are for post-hoc group comparison results from type 2 ANOVAs shown in Supplementary Table 2. Results of the GLM (adjusting for age, sex and education) with subsequent Type 2 ANOVA and least squares difference post-hoc tests (with Holm correction for family of three).
AD=Alzheimer's disease; DLB=dementia with Lewy bodies; HC=healthy control; SD=standard deviation
*$P < 0.05$

Supplementary Figure 1 Cortical thickness group comparisons

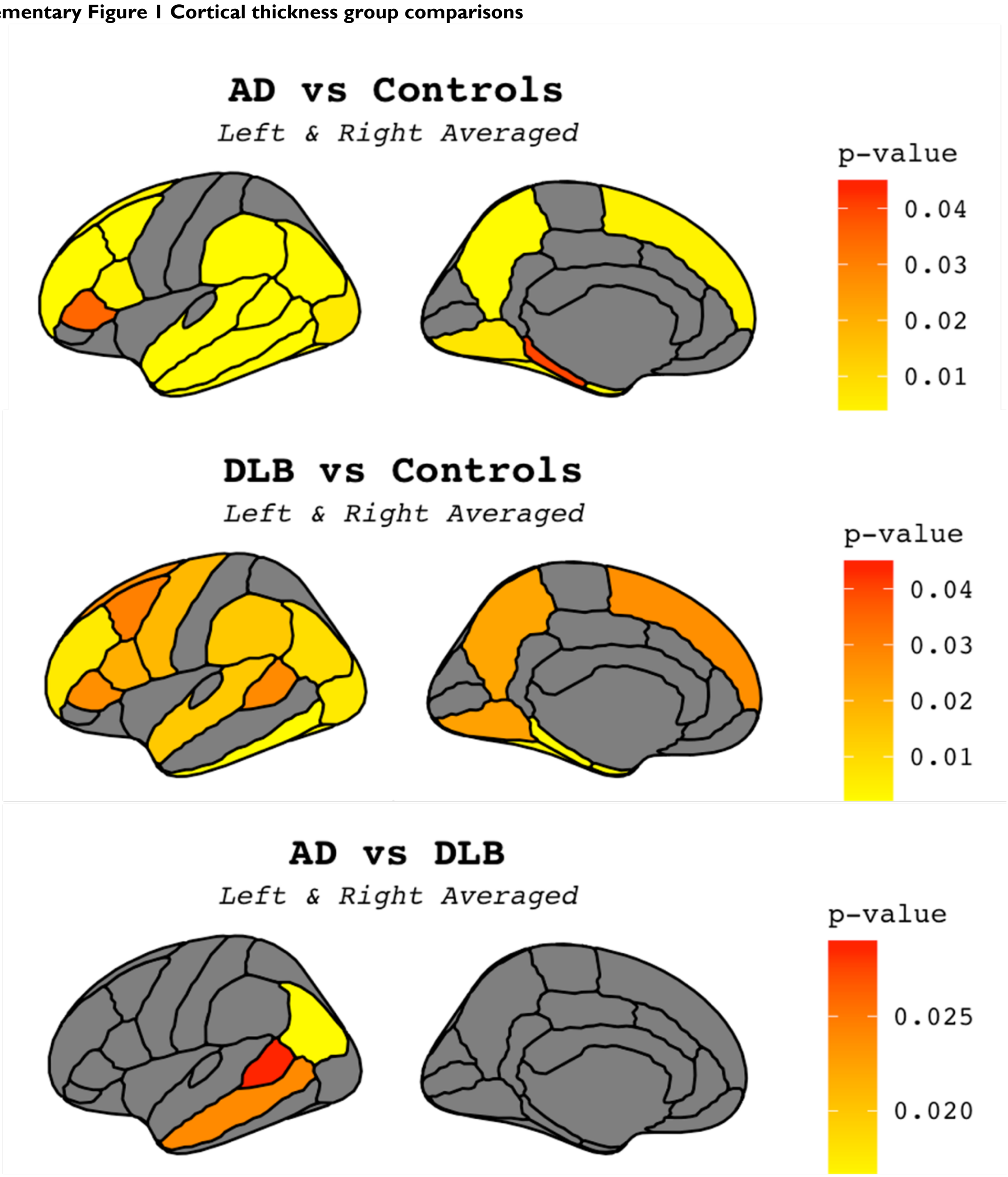

Cortical thickness group comparisons. Highlighted regions were found to be significantly thinner in AD than controls (top), in DLB than controls (middle), and in AD than DLB (bottom). No regions were significantly thinner in DLB than AD. Values are for right and left averaged and projected onto a left hemisphere for graphical representation. P values are for least squares difference post-hoc tests with Holm correction (from GLM and subsequent Type 2 ANOVA reported in Supplementary Table 2. Figure created using the ggseg package [5] in R. AD=Alzheimer's disease; DLB=dementia with Lewy bodies.

Supplementary Table 4 Group comparisons for subcortical region volumes

	AD mean (SD) n=25	DLB mean (SD) n=20	HC mean (SD) n=19	Group difference	Effect size η_p^2
Total intracranial volume	$1.29x10^6$ ($2.13x10^5$)	$1.32x10^6$ ($2.20x10^5$)	$1.21x10^6$ ($1.79x10^5$)	1.18	.04
Subcortical volumes:					
Thalamus	$1.09x10^4$ ($9.72x10^2$)	$1.16x10^4$ ($1.37x10^3$)	$1.16x10^4$ ($1.14x10^3$)	**3.29***[c]	**.10**
Caudate	$6.05x10^3$ ($7.39x10^2$)	$5.88x10^3$ ($9.35x10^2$)	$6.51x10^3$ ($1.39x10^3$)	2.20	.07
Putamen	$7.03x10^3$ ($1.26x10^3$)	$7.00x10^3$ ($1.61x10^3$)	$8.36x10^3$ ($1.43x10^3$)	**4.46***[ab]	**.14**
Pallidum	$3.86x10^3$ ($4.38x10^2$)	$4.10x10^3$ ($5.77x10^2$)	$3.92x10^3$ ($5.35x10^2$)	1.09	.04
Nucleus accumbens	$6.63x10^2$ ($1.23x10^2$)	$6.99x10^2$ ($1.76x10^2$)	$8.31x10^2$ ($2.21x10^2$)	**3.82***[ab]	**.12**
Anterior cingulate	$7.91x10^2$ ($1.70x10^2$)	$8.52x10^2$ ($1.53x10^2$)	$8.88x10^2$ ($1.83x10^2$)	1.51	.05
Mid-anterior cingulate	$4.00x10^2$ ($9.43x10^1$)	$4.23x10^2$ ($9.10x10^1$)	$4.49x10^2$ ($1.10x10^2$)	0.92	.03
Central cingulate	$3.85x10^2$ ($8.00x10^1$)	$4.40x10^2$ ($1.00x10^2$)	$4.21x10^2$ ($7.67x10^1$)	2.11	.07
Mid-posterior cingulate	$3.96x10^2$ ($9.10x10^1$)	$4.72x10^2$ ($1.30x10^2$)	$4.40x10^2$ ($9.47x10^1$)	2.95	.09
Posterior cingulate	$8.96x10^2$ ($2.65x10^2$)	$9.30x10^2$ ($2.39x10^2$)	$1.02x10^3$ ($1.12x10^2$)	0.51	.02
Ventral diencephalon	$6.76x10^3$ ($5.54x10^2$)	$6.96x10^3$ ($9.62x10^2$)	$7.10x10^3$ ($7.98x10^2$)	0.62	.02
Brainstem	$1.84 x10^4$ ($2.03x10^3$)	$1.93x10^4$ ($2.75x10^3$)	$1.88x10^4$ ($1.97x10^3$)	1.18	.04

Group comparisons for subcortical region volumes (left and right volumes combined in mm^3). Results of the GLM (adjusting for age, sex education and total intracranial volume) with subsequent Type 2 ANOVA and least squares difference post-hoc tests (with Holm correction for family of three). AD=Alzheimer's disease; DLB=dementia with Lewy bodies; HC=healthy control; SD=standard deviation
[a]significant post hoc group difference AD-HC
[b]significant post hoc group difference DLB-HC
[c]significant post hoc group difference AD-DLB
*$P < 0.05$
(Note that volumes of whole amygdala and hippocampus are not included here, they are examined alongside segmentation of their respective subfields)

Supplementary Table 5 Mean % difference in subcortical region volumes

	AD % difference (vs HC)	DLB % difference (vs HC)	AD % difference (vs DLB)
Thalamus	-10.3 [-4.2 to -15.8]	-7.5 [-0.2 to -14.7]	**-3.0* [6.5 to -10.5]**
Caudate	-10.2 [-2.5 to -18.1]	-15.5 [-7.9 to -23.2]	6.2 [18.4 to -4.8]
Putamen	**-19.0* [-11.5 to -26.8]**	**-21.2* [-11.5 to -30.9]**	2.8 [20.6 to -10.9]
Pallidum	-6.5 [-0.2 to -12.9]	-3.3 [5.3 to -11.5]	-3.3 [6.6 to -11.3]
Nucleus accumbens	**-23.4* [-12.8 to -33.2]**	**-21.6* [-8.3 to -33.6]**	-2.2 [15.4 to -16.1]
Ventral diencephalon	-9.6 [-2.8 to -15.5]	-9.1 [-0.2 to -17.8]	-0.5 [10.3 to -9.9]
Posterior cingulate	-16.8 [-4.7 to -28.1]	-14.4 [-2.3 to -25.8]	-2.9 [17.0 to -18.3]
Middle posterior cingulate	-14.8 [-1.4 to -25.8]	-1.9 [14.5 to -15.3]	-13.2 [1.8 to -26.1]
Central cingulate	-13.4 [-1.9 to -23.1]	-4.4 [7.9 to -14.9]	-9.4 [2.8 to -20.8]
Middle anterior cingulate	-15.1 [-2.9 to -25.4]	-11.4 [4.4 to -24.3]	-4.2 [13.4 to -18.9]
Anterior cingulate	-15.5 [-4.5 to -25.0]	-10.8 [0.9 to -21.2]	-5.3 [8.6 to -16.4]
Brainstem	-7.3 [0.4 to -14.3]	-5.5 [3.3 to -13.9]	-2.0 [8.2 to -11.3]

Mean % difference in subcortical volumes (as proportion of TIV) (left and right volumes combined). AD=Alzheimer's disease; DLB=dementia with Lewy bodies; HC=healthy control. Significance values are for post-hoc group comparison results from type 2 ANOVAs in table ST4.
*$P < 0.05$

Supplementary Table 6 Group comparisons for hippocampal and amygdala subfield volumes

		AD mean (SD) n=25	DLB mean (SD) n=20	HC mean (SD) n=19	Group difference	Effect size η_p^2
Hippocampus	Whole hippocampus	5015.28 (733.38)	5503.04 (906.09)	6114.47 (930.97)	**9.04***[a]	**.24**
	Hippocampal subfields:					
	CA1	1002.09 (155.52)	1072.42 (184.78)	1176.39 (204.07)	**4.97***[a]	**.15**
	CA2/3	313.17 (45.69)	350.35 (60.08)	385.01 (69.99)	**8.28***[a]	**.23**
	CA4	368.64 (48.41)	403.89 (65.29)	442.63 (63.28)	**8.72***[a]	**.23**
	MLHP	801.73 (124.45)	881.35 (156.57)	996.41 (164.47)	**9.14***[ab]	**.24**
	GCMLDG	421.17 (56.81)	462.34 (76.22)	505.81 (73.96)	**8.76***[a]	**.24**
	Subiculum	621.12 (112.13)	679.13 (146.46)	795.96 (125.17)	**8.36***[ab]	**.23**
	Presubiculum	403.00 (72.38)	444.19 (86.61)	503.87 (78.98)	**7.72***[ab]	**.21**
	Parasubiculum	110.92 (35.77)	118.43 (24.60)	120.73 (20.30)	1.00	.03
	HATA	81.58 (16.17)	92.88 (20.06)	103.95 (17.54)	**7.74***[a]	**.21**
	Fimbria	93.46 (31.73)	107.45 (32.56)	116.19 (29.66)	**3.33***	**.10**
	Tail	798.42 (125.33)	890.61 (135.52)	967.53 (163.16)	**6.16***[a]	**.18**
	Fissure	317.34 (51.19)	319.59 (66.38)	332.44 (53.94)	0.60	.02
	Entorhinal cortex	2531.28 (535.16)	2680.30 (533.31)	3168.58 (720.86)	**5.56***[ab]	**.16**
Amygdala	Whole amygdala	2713.60 (341.29)	2913.95 (494.56)	3202.43 (417.75)	**9.59***[a]	**.25**
	Amygdala subfields:					
	Accessory Basal nucleus	370.54 (54.98)	410.18 (84.39)	471.63 (79.63)	**11.81***[ab]	**.29**
	Anterior amygdaloid area	84.09 (11.05)	89.41 (13.58)	98.76 (13.60)	**9.62***[ab]	**.25**
	Central nucleus	66.71 (13.43)	70.05 (17.43)	85.98 (15.35)	**11.00***[ab]	**.28**
	Medial nucleus	32.34 (6.50)	33.30 (6.88)	41.56 (8.80)	**10.59***[ab]	**.27**
	Cortical nucleus	36.46 (5.40)	39.77 (8.42)	47.06 (8.77)	**11.11***[ab]	**.28**
	CATA	267.29 (36.37)	293.04 (48.27)	308.52 (44.73)	**5.09***[a]	**.15**
	Paralaminar nucleus	87.71 (12.11)	89.14 (16.41)	94.36 (9.87)	1.61	.05
	Lateral nucleus	1070.88 (132.41)	1147.21 (188.01)	1237.69 (141.45)	**8.59***[a]	**.23**
	Basal nucleus	697.58 (92.36)	741.84 (136.26)	816.87 (109.91)	**7.89***[a]	**.22**

Group comparisons for whole hippocampal, whole amygdala and respective subfield volumes (left and right volumes combined). Results of the GLM (adjusting for age, sex, education and total intracranial volume) with subsequent Type 2 ANOVA and least squares difference post-hoc tests (with Holm correction for family of three). AD=Alzheimer's disease; DLB=dementia with Lewy bodies; HC=healthy control; SD=standard deviation; CA= cornu Ammonis; MLHP= molecular layer hippocampus proper; GCMLDG= granule cells of the molecular layer and dentate gyrus; HATA= hippocampal-amygdala transition area; CATA= Cortico-amygdaloid transition area

[a]significant post hoc group difference AD-HC

[b]significant post hoc group difference DLB-HC

*$P < 0.05$

Supplementary Figure 2 Hippocampal (top) and amygdala (bottom) subfield volume group comparisons

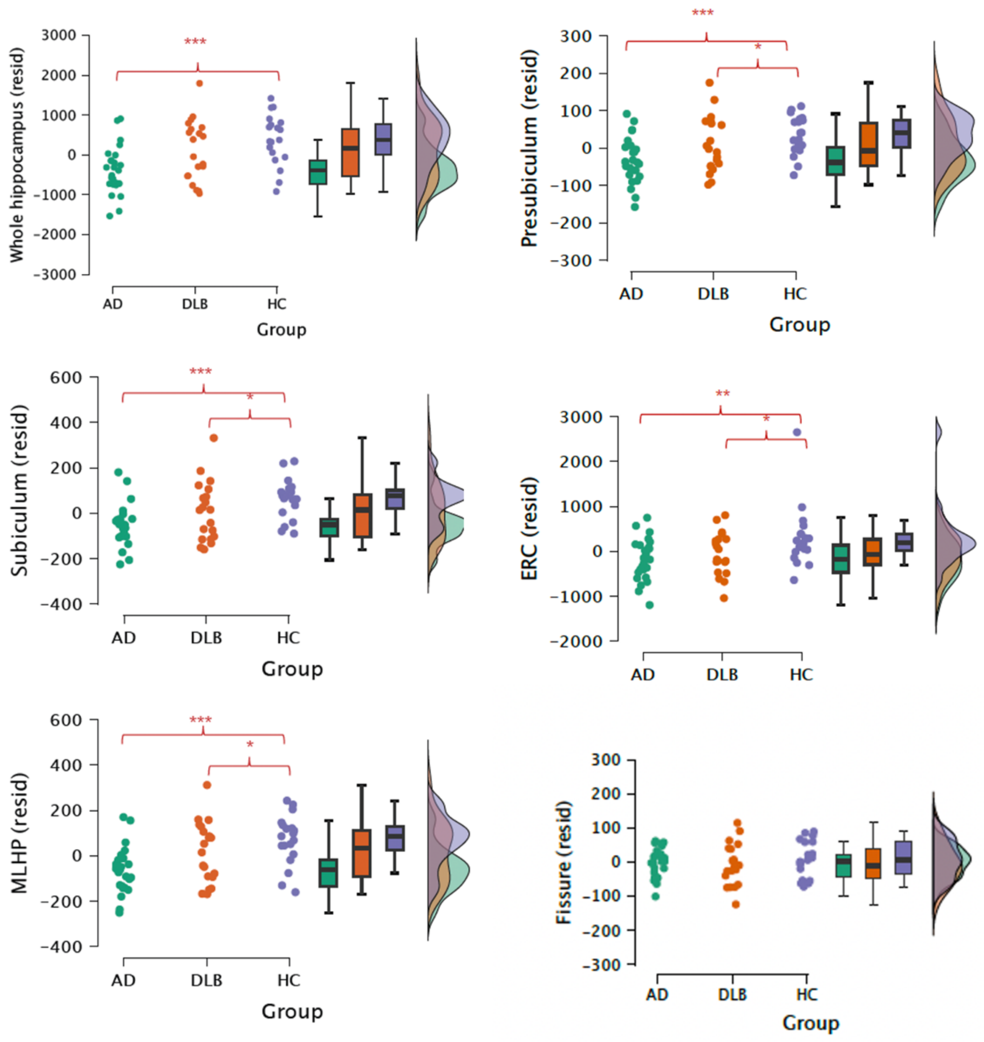

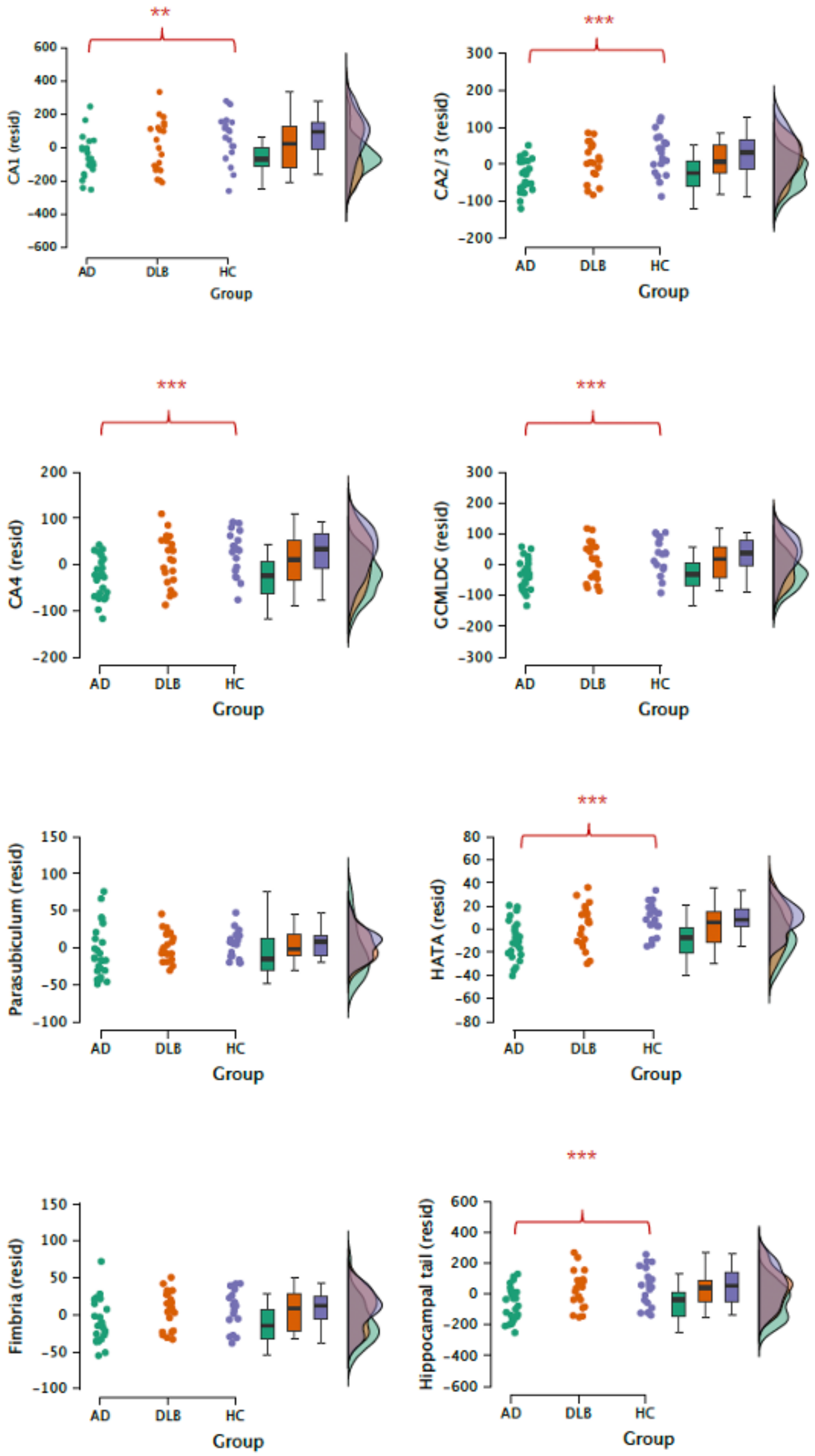

**
CA1 (resid)
600
400
200
0
−200
−400
−600
AD
DLB
HC
Group
***
CA2 / 3 (resid)
300
200
100
0
−100
−200
AD
DLB
HC
Group
***
CA4 (resid)
200
100
0
−100
−200
AD
DLB
HC
Group
***
GCMLDG (resid)
300
200
100
0
−100
−200
−300
AD
DLB
HC
Group
Parasubiculum (resid)
150
100
50
0
−50
−100
AD
DLB
HC
Group
***
HATA (resid)
80
60
40
20
0
−20
−40
−60
−80
AD
DLB
HC
Group
Fimbria (resid)
150
100
50
0
−50
−100
AD
DLB
HC
Group
***
Hippocampal tail (resid)
600
400
200
0
−200
−400
−600
AD
DLB
HC
Group

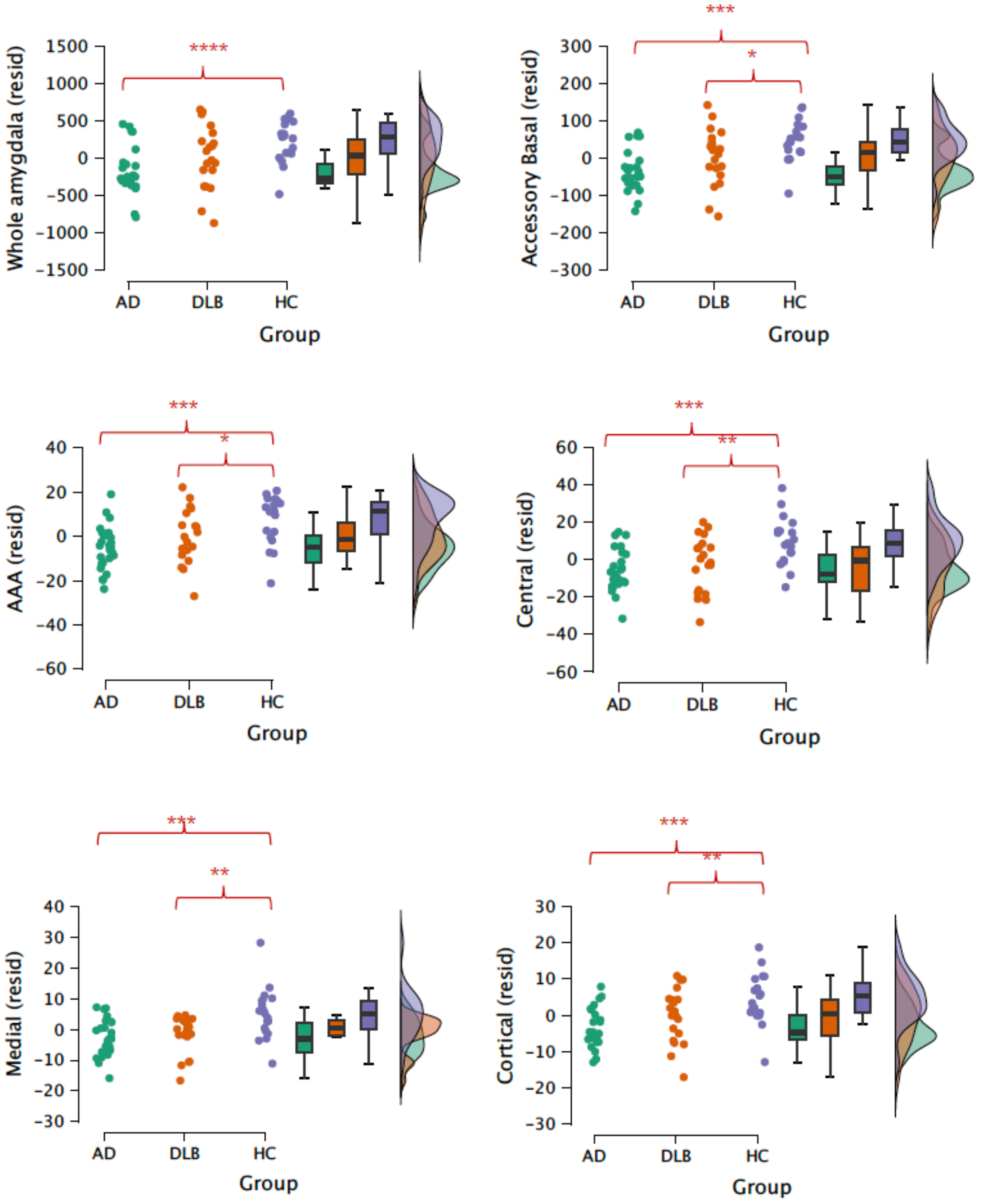

Whole amygdala (resid)
Accessory Basal (resid)
AAA (resid)
Central (resid)
Medial (resid)
Cortical (resid)
AD
DLB
HC
Group
****
***
*
**

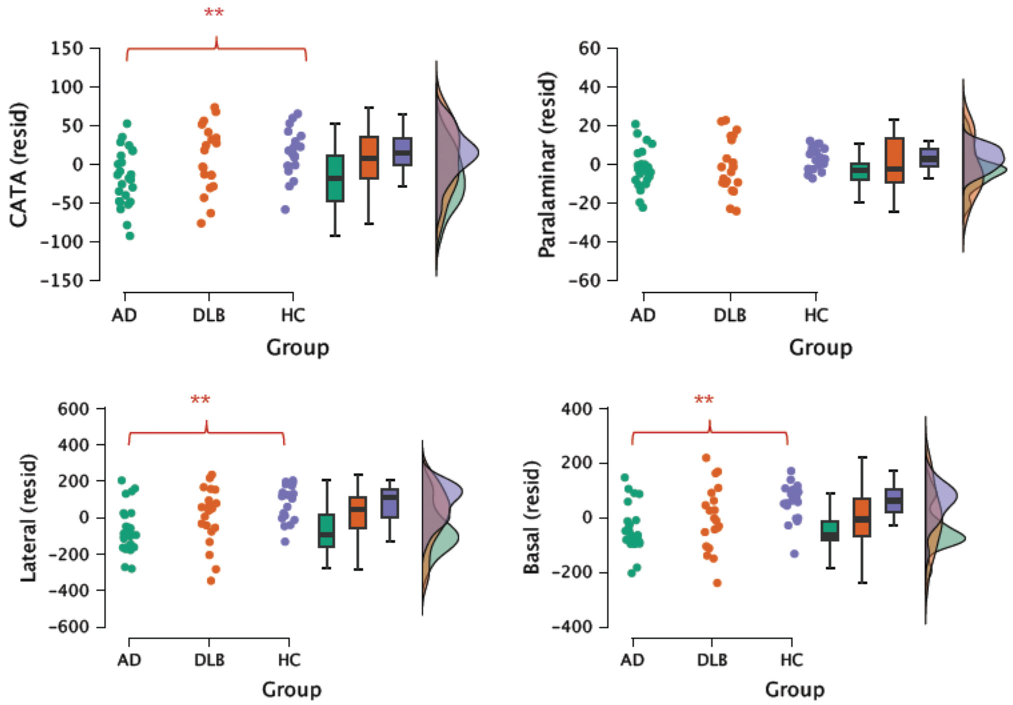

Raincloud plots of hippocampal subfield volumes (top) and amygdala subfield volumes (bottom) (left and right volumes combined). Values plotted are residuals (resid) from a GLM, therefore adjusted for age, sex, education and total intracranial volume. AD=Alzheimer's disease; DLB=dementia with Lewy bodies; HC=healthy control; MLHP= molecular layer hippocampus proper; ERC= entorhinal cortex; AAA= anterior amygdaloid area; CATA= cortico-amygdala transition area.
Significance *P* <.001***, <.01**, <.05*

Supplementary Figure 4 Scatter plots of whole amygdala volumes with visual hallucinations questionnaire scores

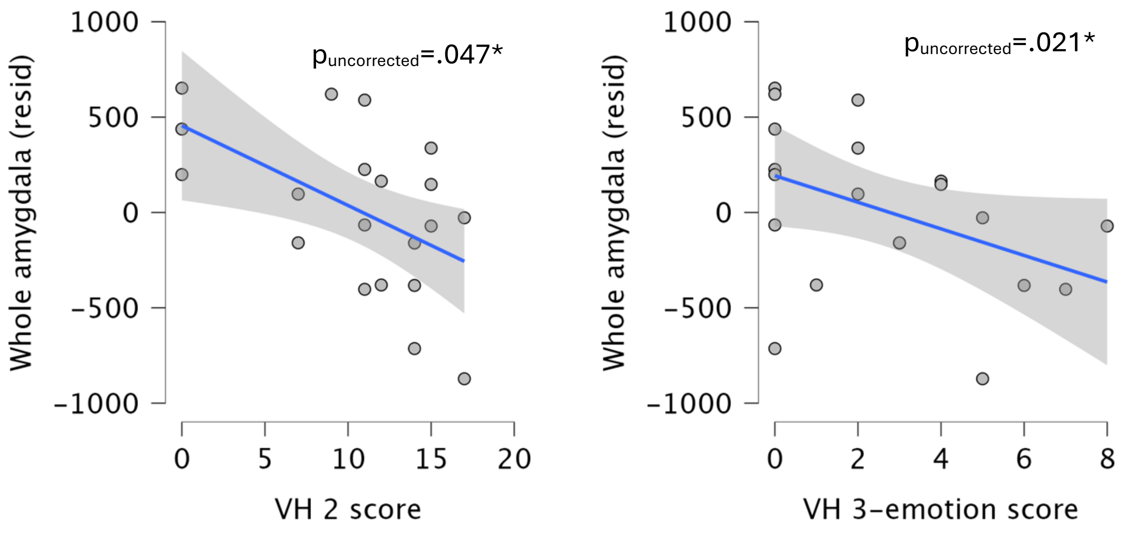

Scatter plots of whole amygdala volumes with visual hallucination questionnaire scores - DLB group only (left and right volumes combined). Plotted values are residuals (resid) - adjusted for age, sex, education, and total intracranial volume. VH 2= Visual hallucinations part 2 score (frequency and duration); VH 3-emotion= Visual hallucinations part 3- emotions sub-score right

Significance (uncorrected) P <.05*

Supplementary Table 7 Correlations for amygdala and subfield volumes (left and right volumes combined) with visual hallucinations part 2 (frequency and duration) and part 3 (emotional response) scores (dementia with Lewy bodies group only) – using imputed values for missing data, with additional covariate of ACE-III to control for disease severity.

	VH part 2 score	VH part 3 score
	Kendall's tau	
Whole amygdala	-0.137	**-0.358***
Subfields		
Lateral nucleus	0.074	**-0.337***
Basal nucleus	-0.179	**-0.358***
Accessory Basal nucleus	-0.126	-0.221
AAA	-0.179	-0.316
Central nucleus	0.137	-0.000
Medial nucleus	0.095	-0.000
Cortical nucleus	-0.074	-0.295
CATA	-0.232	-0.242
Paralaminar nucleus	0.084	**-0.389***

Plotted values are residuals adjusted for age, sex, education, total intracranial volume, and ACE-III
AAA= anterior amygdaloid area; CATA= cortico-amygdala transition area; VH=visual hallucinations
*P < 0.05
[a]survives correction for multiple comparisons with false discovery rate
Values in red are different in significance compared to the original analysis shown in Table 4.

Supplementary Table 8 Comparison of raw whole hippocampal and whole amygdala volumes between patient categories in plasma ptau-217 sub-group:

	AD	DLB-	DLB+
**n**	17	4	9
**Whole hippocampus**	4958.19 (661.12)	6040.92 (542.88)	5290.96 (740.59)
**Whole amygdala**	2671.34 (335.22)	3265.18 (303.60)	2846.62 (360.33)

Mean (standard deviation) by group.

Key: AD=Alzheimer's disease; DLB=dementia with Lewy bodies; DLB+/-=DLB with ptau-217 above/below the threshold suggested in [6]

Supplementary Figure 5 Raincloud plots for raw whole hippocampal and whole amygdala volumes between patient categories in plasma ptau-217 sub-groups:

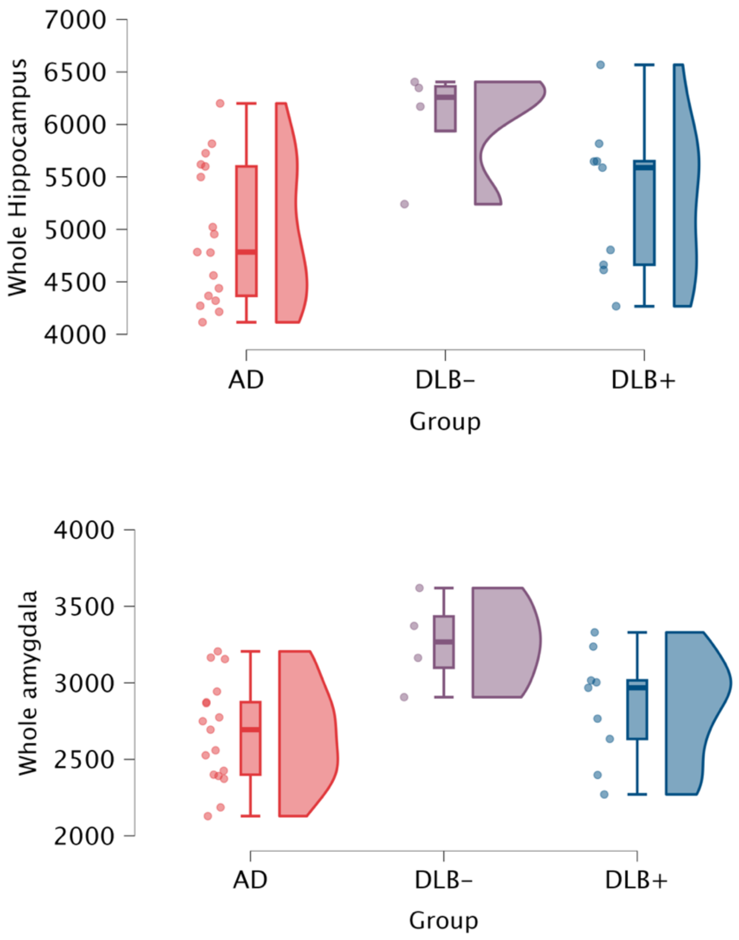

Key: AD=Alzheimer's disease; DLB=dementia with Lewy bodies; DLB+/-=DLB with ptau-217 above/below the threshold suggested in [6]

Power calculation

Power calculations using information from published studies was performed using the Basic Functions for Power Analysis (pwr) package [7] in R (v3.5.3 [8]). At the time of writing the study protocol (in 2017) there were no published 7T studies in DLB to properly inform a power calculation. Therefore, sample size was calculated based on data from DLB MRI at lower field strengths and 7T MRI studies of AD. Based on the initial power calculations we aimed to recruit 24 participants per group.

Power calculation was repeated in 2020 early in recruitment for the main body of the study. A significant difference in right and left cornu Ammonis- stratum radiatum/stratum lacunosum-moleculare (SRLM) volumes was reported in AD compared to HC at 7T [9]. On the right cross-sectional area of the SRLM for AD (n=4) was 5.6mm2 +/-0.5 and HC (n=7) 9.2mm2 +/-2.2. Using a 2-sided test, alpha 0.05 and power 0.9, 6 participants would be required in each group. On the left cross-sectional area of the SRLM for AD was 5.7 mm2 +/-1.2 and HC 7.9mm2 +/-1.3. Using a 2-sided test, alpha=0.05 and power=0.9, 8 participants would be required in each group.

The effect size from this 7T study suggested that fewer participants than previously planned may be necessary for an adequately powered analysis. However, these studies (and the majority of studies published thus far using 7T MRI) are extremely small and potentially underpowered, therefore, any significant effect sizes are likely to be inflated [10]. The revised recruitment goal was 20 participants per group.

QC examples:

Skull-stripping: Conservative (rather than strict) removal of skull and dura produced best results in the following segmentation steps. Below is a typical example of the extent of stripping used in the structural pipeline:

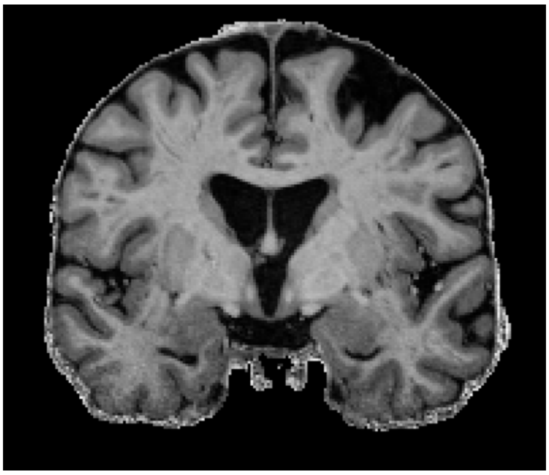

Recon-all segmentation: No segmentations failed to align accurately. Most "poor" segmentation was in the cerebellum. Since this was not a region of interest, scans with poor segmentation in this region were not excluded. Below is a typical example of "poor" segmentation:

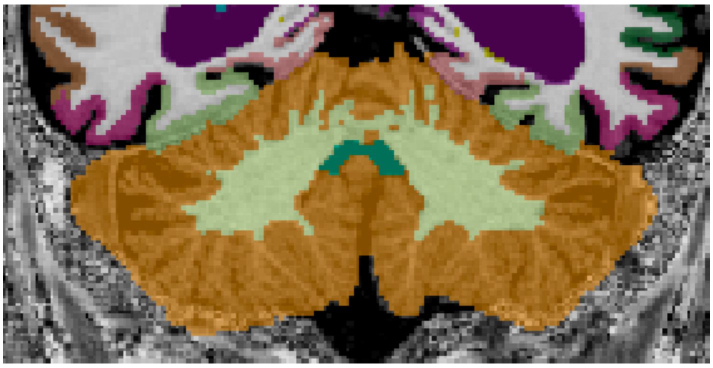

Below is a typical example of "good" segmentation:

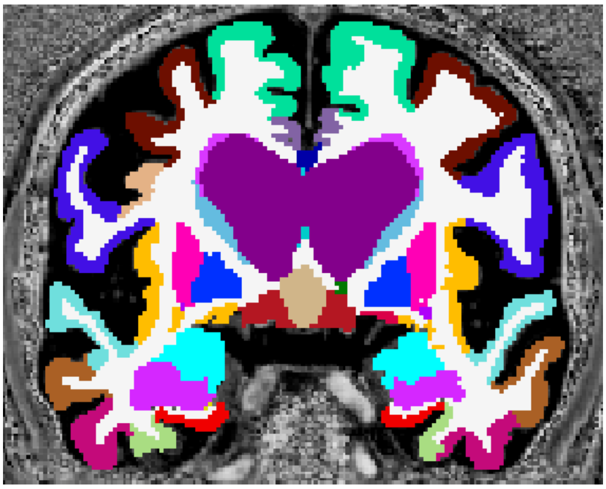

Medial temporal lobe structures of interest:

<table>
<tr><td rowspan="21">Medial temporal lobe</td><td></td><td></td><td>Subfield</td><td>Acronym</td></tr>
<tr><td rowspan="12">Whole hippocampus</td><td rowspan="7">Hippocampus proper</td><td>Cornu Ammonis 1</td><td>CA1</td></tr>
<tr><td>Cornu Ammonis 2/3</td><td>CA2/3</td></tr>
<tr><td>Cornu Ammonis 4</td><td>CA4</td></tr>
<tr><td>Molecular layer of the hippocampus proper</td><td>MLHP</td></tr>
<tr><td>Granule cells/molecular layer/dentate gyrus</td><td>GCMLDG</td></tr>
<tr><td>Fimbria</td><td></td></tr>
<tr><td>Hippocampal amygdala transition area</td><td>HATA</td></tr>
<tr><td rowspan="3">Subicular cortex</td><td>Subiculum</td><td></td></tr>
<tr><td>Presubiculum</td><td></td></tr>
<tr><td>Parasubiculum</td><td></td></tr>
<tr><td colspan="2">Hippocampal tail</td><td></td></tr>
<tr><td colspan="2">Hippocampal fissure</td><td></td></tr>
<tr><td colspan="3">Entorhinal cortex</td><td>ERC</td></tr>
<tr><td rowspan="7">Whole Amygdala</td><td colspan="2">Accessory Basal nucleus</td><td></td></tr>
<tr><td colspan="2">Anterior amygdaloid area</td><td>AAA</td></tr>
<tr><td colspan="2">Central nucleus</td><td></td></tr>
<tr><td colspan="2">Medial nucleus</td><td></td></tr>
<tr><td colspan="2">Cortical nucleus</td><td></td></tr>
<tr><td colspan="2">Cortico-amygdaloid transition area</td><td>CATA</td></tr>
<tr><td colspan="2">Paralaminar nucleus</td><td></td></tr>
</table>

Additional citations:

1. Sheikh JI, Yesavage JA. 9/geriatric depression scale (Gds) recent evidence and development of a shorter version. Clin Gerontol. 1986;5:165–73. https://doi.org/10.1300/J018v05n01_09

2. Fahn S, Elton R. Recent developments in Parkinsons disease. Fahn S, Marsden C, Goldstein M CD, editors. New Jersey: Macmillan Healthcare Information; 1987.

3. Walker MP, Ayre GA, Cummings JL, Wesnes K, McKeith I G, O'Brien JT, Ballard CG. The Clinician Assessment of Fluctuation and the One Day Fluctuation Assessment Scale. British Journal of Psychiatry, 2000:177(3), 252–256. doi: 10.1192/bjp.177.3.252. PMID: 11040887

4. Thomas AJ, Taylor JP, McKeith I, Bamford C, Burn D, Allan L, O'Brien, JT. Development of assessment toolkits for improving the diagnosis of the Lewy body dementias: feasibility study within the DIAMOND Lewy study. Int J Geriatr Psychiatry. John Wiley and Sons Ltd; 2017;32:1280–304. https://doi.org/10.1002/gps.4609

5. Mowinckel AM, Vidal-Piñeiro D. Visualization of Brain Statistics With R Packages *ggseg* and *ggseg3d*. Adv Methods Pract Psychol Sci. 2020;3:466–83. https://doi.org/10.1177/2515245920928009

6. Ashton NJ, Brum WS, Molfetta G Di, Benedet AL, Arslan B, Jonaitis E, Langhough RE, Cody K, Wilson R, Carlsson CM, Vanmechelen E, Montoliu-Gaya L, Lantero-Rodriguez J, Rahmouni N, Tissot C, Stevenson J, Servaes S, Therriault J, Pascoal T, Lleó A, Alcolea D, Fortea J, Rosa-Neto P, Johnson S, Jeromin A, Blennow K, Zetterberg H. Diagnostic Accuracy of a Plasma Phosphorylated Tau 217 Immunoassay for Alzheimer Disease Pathology. JAMA Neurol. 2024;81:255–63. https://doi.org/10.1001/jamaneurol.2023.5319

7. Champely, S. pwr: Basic Functions for Power Analysis. 2020 (R package version 1.3-0).

8. R Core Team. R: A language and environment for statistical computing [Internet]. URL https://www.R-project.org/. Vienna, Austria: R Foundation for Statistical Computing; 2023. https://www.R-project.org/. Accessed 21 Apr 2023

9. Boutet C, Chupin M, Lehéricy S, Marrakchi-Kacem L, Epelbaum S, Poupon C, Wiggins C, Vignaud A, Hasboun D, Defontaines B, Hanon O, Dubois B, Sarazin M, Hertz-Pannier L, Colliot O. Detection of volume loss in hippocampal layers in Alzheimer's disease using 7 T MRI: A feasibility study. Neuroimage Clin. Elsevier Inc.; 2014;5:341–8. https://doi.org/10.1016/j.nicl.2014.07.011

10. Button KS, Ioannidis JPA, Mokrysz C, Nosek BA, Flint J, Robinson ESJ, Munafò MR. Power failure: Why small sample size undermines the reliability of neuroscience. Nat Rev Neurosci. 2013;14:365–76. https://doi.org/10.1038/nrn3475
